# Mannose and phenylboronate ester functionalized mesoporous silica nanoparticles contained in chitosan microneedles for enhancing cellular immunity and antitumor efficacy

**DOI:** 10.7150/thno.121610

**Published:** 2026-01-01

**Authors:** Nanxi Chen, Ye He, Yuxuan Zhang, Yong Cui, Hongyan Lu, Jinghai Zhang, Haotian Zhang, Qinfu Zhao, Yuling Mao, Yikun Gao, Siling Wang

**Affiliations:** 1 Department of Pharmaceutics, School of Pharmacy, Shenyang Pharmaceutical University, Shenyang 110016, China.; 2 Department of Biomedical Engineering, School of Medical Devices, Shenyang Pharmaceutical University, Shenyang 110016, China.; 3 Department of Pharmacology, Shenyang Pharmaceutical University, Shenyang 110016, China.

**Keywords:** cancer immunotherapy, intracellular protein delivery, chitosan microneedle, adjuvant, cellular immunity

## Abstract

**Rationale**: Immunotherapy has emerged as a crucial component in cancer treatment, particularly for the long-term reduction of cancer metastasis and recurrence. However, its development is hindered by limited activation of cellular immune response and suboptimal delivery of vaccine to antigen-presenting cells.

**Methods**: The vaccine was encapsulated within mesoporous silica nanoparticles, followed by functionalization by mannose and phenylboronate ester (MSN-NH-DPM), which facilitates targeting antigen-presenting cells via mannose receptors and enables intracellular delivery through endosomal escape, thereby activating cellular immunity. The nanoparticles were then integrated into chitosan microneedle patches (MNs), which are engineered to deliver the nanoparticles into the skin that is abundant in immune cells, and improve the immune response through the adjuvant properties of chitosan.

**Results**: The chitosan MNs incorporating MSN-NH-DPM (CTS-MN@MSN-NH-DPM) significantly activated the cellular immune response through the MHC-I pathway. The antigen-presenting cells that uptake the vaccine migrated to nearby lymph nodes, inducing systemic immunity to eliminate cancer cells. Compared with subcutaneous injection, the application of CTS-MN@MSN-NH-DPM significantly inhibited the growth of B16/OVA melanoma tumors and extended the survival time of the melanoma mouse model.

**Conclusions**: The MNs with targeted and intracellular delivery represent a promising platform for various vaccines to improve the cellular immune response, thus providing a potential solution for cancer treatment.

## Introduction

The accessibility and affordability of high-throughput sequencing technologies enable the identification of numerous tumor neoantigens. Concurrently, advancements in protein engineering have redirected the focus of cancer vaccines from entirely inactivated or attenuated pathogens to subunit vaccines. These subunit vaccines consist of polypeptides composed of known or predicted tumor antigen epitopes, which demonstrate high specificity and potential efficacy 1. Unfortunately, the constraints of major histocompatibility complex (MHC) polymorphism and the restricted number of antigenic epitopes limit the immunogenicity of peptide-based vaccines 2. Furthermore, cellular immunity is vital for combating cancers 3,4, and dendritic cells (DCs) must present antigens through the MHC class I (MHC-I) pathway to trigger a robust CD8^+^ T-cell response 5. This process necessitates that the antigen be either delivered to the classical cytosolic MHC-I antigen processing pathway or endocytosed by specific cross-presenting DCs. However, peptide-based vaccines are susceptible to getting trapped in lysosomes and being degraded, thereby blocking this process 6,7. Therefore, the effective intracellular delivery of cancer vaccines is crucial for inducing robust cellular immunity to combat cancer cells.

In recent years, researchers have suggested various polymers for facilitating intracellular protein delivery 8, including cell-penetrating peptides 9,10, phenylboronic acid 11,12, arginine 13,14, and poly(disulfide)s 15,16. These polymers transport proteins to the cytoplasm through mechanisms such as endosomal escape and membrane translocation. However, they are limited by the uptake efficiency of immune cells and are rarely used to improve the cellular immunity elicited by vaccines. In particular, phenylboronate ester could achieve endosomal escape and possess a structure as a bridging agent. Phenylboronate ester formed by catechol and borate is acid-labile, and the acidic conditions of endosomes break the linkage to release phenylboronic acid 17. Subsequently, boric acid interacts with endosomal membranes, thereby compromising their stability and facilitating endosomal escape 18. Mannose can be endocytosed more by immune cells because it can be recognized by mannose receptors on immune cells 19-21. Therefore, it is anticipated that the combination of phenylboronate ester and mannose would solve the problem of less uptake of phenylboronate ester by immune cells and increase the amount of nanoparticles for endosomal escape to reach the cytoplasm. Additionally, mesoporous silica nanoparticles (MSNs) are particularly effective in the stable loading of vaccines, protecting them from enzymatic degradation and allowing for facile modification 22,23. Hence, we anticipate that the simultaneous modifications of mannose for promoting cellular uptake and phenylboronate ester for endosomal escape on MSNs could effectively improve their efficacy as carriers for cancer vaccine delivery and trigger more robust immune responses to impede cancer progression.

The skin functions as an “autonomous lymphoid organ”, capable of independently producing antibodies even in the absence of secondary lymphoid organs, and it is a highly conductive organ, where the application of bacteria to the surface can stimulate nearby lymph nodes (LNs) to form germinal centers and produce corresponding antibodies 24. The abundant antigen-presenting cells (APCs) of the skin, including DCs, Langerhans cells, macrophages, and neutrophils, improve the ability of vaccine to provoke a strong immune response 25. Some studies suggested that transdermal vaccine delivery triggered more robust immune responses than larger doses by deep subcutaneous injection 26. Hence, MNs have been used in cancer treatment 27,28. MNs also possess several advantages, including being minimally invasive 29-31 and painless 32,33, reduced difficulties caused by needle-based administration 34, and decreased reliance on cold chain infrastructure 35. Moreover, MNs possess adequate capacity to load nanoparticles, thereby facilitating the intradermal delivery of particle carriers. Chitosan, a frequently used substrate for MNs, not only dries and solidifies easily but also exhibits adjuvant properties that can improve the immune response 36. Consequently, chitosan MNs represent a promising tool for boosting the efficacy of vaccines as well.

**Figure 1:**
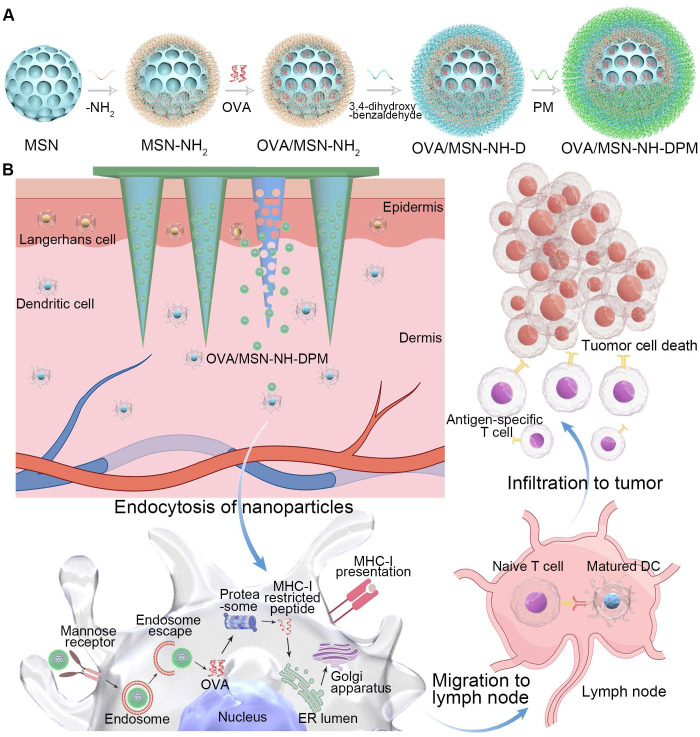
Schematic of vaccine intracellular delivery platform (MSN-NH-DPM) construction and delivery *in vivo* after microneedle vaccination. (**A**) Construction of MSN-NH-DPM. (**B**) Intracellular delivery of OVA/MSN-NH-DPM after CTS-MOM vaccination, inducing cellular immunity and combating cancer cells.

In this study, we innovatively constructed chitosan MNs loaded with MSN-NH-DPM (Figure 1A). Ovalbumin (OVA) was selected as the model antigen. MSNs were specifically engineered to facilitate the efficient and stable encapsulation of OVA (OVA/MSN). The compound 4-aminophenylboronic acid was covalently bonded with mannose to form a Schiff base derivative (PM) and subsequently conjugated with the carrier through 3,4-dihydroxybenzaldehyde to obtain the functional complex (OVA/MSN-NH-DPM). Chitosan was mixed with polyvinylpyrrolidone (PVP) to serve as the MN substrate. Then, OVA/MSN-NH-DPM was incorporated into the substrate and poured into an MN mold. After drying and demolding, MNs containing OVA/MSN-NH-DPM (MOM) were successfully obtained. After inserting the chitosan MOM (CTS-MOM) into the skin, chitosan substrates gradually dissolved to improve the immune response and release OVA/MSN-NH-DPM (Figure 1B). Subsequently, the complexes were uptaken by immune cells via mannose receptor and successfully escaped from endosomes to reach the cytoplasm due to the phenylboronate ester, and then OVA was released and presented through the MHC-I pathway to facilitate cellular immune response. These immune cells migrated to the nearby LNs, resulting in the induction of cytotoxic T lymphocytes (CTLs), which in turn target and eliminate the corresponding cancer cells. This strategy, which further increases the intracellular delivery efficiency through delivery to immune cell-rich regions and by targeting ligands, improves the immune response, and breaks the conventional difficulty of subunit vaccines in generating cellular immunity, thereby providing a simple and universal platform for preparing personalized cancer vaccines.

## Materials and Methods

### Materials and animals

Unless mentioned otherwise, chemicals were purchased from Aladdin (Shanghai, China). Polyvinyl pyrrolidone (K90) was purchased from BASF (Ludwigshafen, Germany). Chitosan, with a molecular weight of 300 kDa and a deacetylation degree of 95%, was obtained from SHANDONG AK BIOTECH (Shandong, China). All cell supplies, organelle probes, transport inhibitors, were purchased from Meilun Biotechnology (Dalian, China). All antibodies employed were purchased from Thermo Fisher (CA, USA). Murine granulocyte-macrophage colony-stimulating factor (GM-CSF) was purchased from Biolegend (CA, USA). Female polydimethylsiloxane (PDMS) MN molds were purchased from Taizhou Microchip Pharmaceutical Technology (Taizhou, China). A 10 × 10 array of MNs was possessed with a tip-to-tip distance of 1300 μm. The MNs was shaped like a cone, whose height is 1000 μm and base diameter is 450 μm. To handle more intricate skin conditions, a base with a height of 200 μm and a diameter of 1000 μm was incorporated.

Female C57BL/6 mice, aged 6-8 weeks, were obtained from the Experimental Animal Center at Shenyang Pharmaceutical University. Animal experiments were performed in accordance with the National Research Council's Guide for the Care and Use of Laboratory Animals and approved by the Experimental Ethics Committee of Shenyang Pharmaceutical University (Approval No. SYPU-IACUC-S2024-10.30-102).

### Synthesis and characterization of MSN-NH-DPM

MSNs were synthesized using a customized procedure described previously 37. Briefly, sodium salicylate, cetyltrimethylammonium bromide, and triethanolamine were added in water at a molar ratio of 1:0.8:0.36. After adding 4 mL of tetraethyl orthosilicate to the reaction mixture, it was stirred at 80 °C for 2 h. Then, the mixture was centrifuged to collect the precipitate, and the CTAB template was removed by calcination in a muffle furnace for 6 h at 500 °C to yield MSNs. Amination was used to further modify the synthesized MSNs, obtaining MSN-NH_2_. Through a Schiff base reaction, 3,4-dihydroxybenzaldehyde and MSN-NH_2_ were combined to produce MSN-NH-D. Moreover, mannose and 4-aminophenylboronic acid were reacted to form the Schiff base derivative PM. Next, MSN-NH-D and PM were linked through an ester bond, forming MSN-NH-DPM. To load OVA (OVA/MSN-NH_2_), 5 mL of an OVA aqueous solution (2 mg/mL) was mixed with 5 mL of a dispersed MSN-NH_2_ aqueous solution (1 mg/mL) and subjected to probe sonication for 5 min (150 W, 5 s on, 5 s off) in an ice bath. After centrifugation, the OVA/MSN-NH_2_ was sequentially redispersed into D and PM solutions to form OVA/MSN-NH-DPM. To determine the vaccine loading amount, the Bradford protein assay was used to measure the protein amount not loaded in the supernatant, and the vaccine loading efficiency (LE) was calculated using the following equation:

LE(%) = (OVA_total_ - OVA_free_) ÷ (Carrier_total_ + OVA_total_ - OVA_free_)

where OVA_total_ is the mass of OVA added into the solution, Carrier_total_ is the mass of nanoparticles added into the solution, and OVA_free_ is the mass of free OVA in the supernatant after sonication and centrifugation.

The morphology of the synthesized nanoparticles was examined under a transmission electron microscope (TEM, Tecnai G2 F30, FEI, Eindhoven, Netherlands). Particle size distribution and zeta potential were determined using a Nicomp 380 Particle Size Analyzer (Particle sizing systems, USA). The specific surface area (S_BET_), pore volume (Vt), and pore size distribution (P_D_) of the nanocarriers were determined using a surface area and pore size analyzer (V-Sorb 2800P, Gold APP Instrument Corporation, China). Surface element composition was analyzed by X-ray photoelectron spectroscopy (Thermo ESCALAB 250XI, USA), and the modification amounts of various groups were quantified using a thermogravimetric analyzer (TGA 550, TA Instruments, USA).

### Fabrication and characterization of MOM patch

MNs were produced using the centrifugation approach. In detail, a PVP aqueous solution with a concentration of 0.2 g/mL was added to the PDMS mold and centrifuged at 3773 *g* for 3 min. The liquid outside the pinhole was removed, and the remaining portion was dried at 20 °C for 30 min, after which the PVP solution shrunk and formed a thin shell. Meanwhile, the OVA/MSN-NH-DPM were uniformly dispersed either into a polymer solution (PVP and CTS in a mass ratio of 7:3 with 0.2 g/mL) for CTS-MN@OVA/MSN-NH-DPM (CTS-MOM) or into a PVP solution (0.2 g/mL) for PVP-MN@OVA/MSN-NH-DPM (PVP-MOM). Next, the mixture was filled in the PVP shells in the mold by centrifugation. After removing the liquid outside the pinhole, the amount of drug loading was calculated by weighing the mold before and after adding the mixture. Due to the shrinkage of the mixture after drying, the filling and drying process must be repeated four times until the dried mixture completely filled the cavity. Finally, the PVP solution (0.2 g/mL) was filled above the mixture to form the base to obtain CTS-MOM or PVP-MOM.

### *In vitro* drug release and skin penetration test

The release of OVA from OVA/MSN-NH-DPM, PVP-MOM, and CTS-MOM was tested in phosphate-buffered saline (PBS) at 37 °C. Briefly, 0.5-mL samples were collected at specific time intervals and subjected to centrifugation in 4 °C. The release of OVA was then determined by measuring the protein content in the supernatant. Simultaneously, the precipitates were resuspended in the release system with fresh buffer (equal volume). The concentration of released protein was measured using the Bradford protein assay. The dorsal skin excised from C57BL/6 mice was mounted onto a transdermal diffusion cell. Then, PVP-MOM and CTS-MOM were administered to the skin, and the transdermal release of OVA was quantified as described earlier. Changes in the secondary conformation of OVA encapsulated within or released from OVA/MSN-NH-DPM were analyzed by circular dichroism (CD) spectroscopy (Bio-Logic MOS 450, France). Additionally, the integrity of OVA released from OVA/MSN-NH-DPM, PVP-MOM, and CTS-MOM was determined by sodium dodecyl sulfate-polyacrylamide gel electrophoresis (SDS-PAGE). Furthermore, the potential for drug release at the target site was explored by examining the degradation of the released MSN-NH-DPM in simulated body fluids under a TEM.

The mechanical strength of PVP-MOM and CTS-MOM was evaluated using a push-pull dynamometer (model HP-5, Handpi, China). The plate moved toward the needle tips at a speed of 0.12 mm/s until needle buckling occurred. The dorsal skin of C57 mice was prepared by shaving with an electric clipper and subsequently depilating using a hair-removal cream. PVP-MOM and CTS-MOM were applied to the mice using a custom-made injector. After inserting the MOM patches into the mouse skin, the insertion depth was determined by optical coherence tomography (OCT, VivoSight Dx, Michelson Diagnostics Ltd., UK). Then, the patch was removed to capture photographs. Subsequently, the skin inserted with the MOM patches was stained with 0.4% trypan blue for 1 min and photographed after wiping.

### Intracellular delivery mechanism of the functionalized MSNs

To investigate the endocytosis pathways of the functionalized MSNs, we pretreated DC2.4 cells with amiloride hydrochloride (EIPA), methyl-β-cyclodextrin (Mβ-CD), or chlorpromazine (CPZ) at 37 °C for 1 h. Next, 20 μg/mL of fluorescein isothiocyanate (FITC)-labeled MSN-NH-DPM was added and incubated for an additional 4 h. Then, the cells were washed in PBS, collected, and examined by flow cytometry. For investigating the intracellular distribution, DC2.4 cells (1 × 10^4^ cells/well) were cultured in 24-well plates and incubated with OVA/MSN-NH_2_, OVA/MSN-NH-DP, and OVA/MSN-NH-DPM, each containing 20 μg/mL of FITC-labeled OVA, for 0.5 and 4 h. Then, lysosomes and nuclei were stained with Lysotracker Red and 4′,6-diamidino-2-phenylindole (DAPI), respectively. The localization of nanoparticles within cells was observed by confocal laser scanning microscopy (CLSM).

### Distribution of nanoparticles in mice

MSN-NH-DPM, PVP-MN@MSN-NH-DPM, and CTS-MN@MSN-NH-DPM were constructed using FITC-labeled MSN-NH_2_ according to the above-described method. Then, mice were treated with MSN-NH_2_, MSN-NH-DPM, PVP-MN@MSN-NH-DPM, and CTS-MN@MSN-NH-DPM. All treatments, except for MN, were administered via subcutaneous injection, and the dose of MSN-NH_2_ in each group was 20 mg/kg. At 6, 24, and 48 h, the mice were examined using a Caliper IVIS® Spectrum System (PerkinElmer). Additionally, various organs were isolated at 24 h to observe the internal distribution of nanoparticles within the mice. Furthermore, LNs were harvested at 24 h to quantify the silicon content by inductively coupled plasma mass spectrometry (ICP-MS 7800, Agilent).

To explore whether OVA can be delivered to LNs, OVA/MSN-NH_2_, OVA/MSN-NH-DPM, PVP-MOM, and CTS-MOM were constructed using RBITC-labeled MSN-NH_2_ and FITC-labeled OVA. Then, inguinal LNs were collected for cryosection at 24 h after treatment with OVA, OVA/MSN-NH_2_, OVA/MSN-NH-DPM, PVP-MOM, and CTS-MOM at 20 mg/kg of MSN-NH_2_ in mice. The nuclei were then stained with DAPI in the inguinal LN tissue sections, after which the colocalization of OVA and nanoparticles in inguinal LNs was examined under a fluorescence microscope.

### Pathway of cross-presentation and bone marrow-derived dendritic cell (BMDC) maturation

To examine the cross-presentation of OVA, DC2.4 cells were cultured in a 12-well plate and treated with OVA (10 μg/mL), OVA/MSN-NH (10 μg/mL OVA), OVA/MSN-NH-DP (10 μg/mL OVA), and OVA/MSN-NH-DPM (10 μg/mL OVA) for 24 h. Next, the cells were harvested, washed with PBS, stained with the monoclonal antibody 25d1.16, and examined by flow cytometry. To clarify the pathway used by DC2.4 cells for the cross-presentation of OVA, the cells were pretreated with mannose (100 µg/mL), brefeldin A (BFA, 1 µg/mL), MG-132 (1 µg/mL), or leupeptin (100 µg/L) at 37 °C for 1 h. After pretreatment, OVA/MSN-NH-DPM containing 10 µg of OVA was added, and incubation was continued for an additional 24 h. The cells were then collected, washed with PBS, stained with the monoclonal antibody 25d1.16, and analyzed by flow cytometry.

BMDCs were isolated from mice and cultured in 12-well plates. The cells were then incubated with PBS, OVA (10 μg/ mL), OVA/MSN-NH_2_ (10 μg/mL OVA), OVA/MSN-NH-DPM (10 μg/mL OVA), and lipopolysaccharide (1 μg/mL) for 24 h. The supernatant was collected to measure the secretion levels of TNF-α and IL-12 by enzyme-linked immunosorbent assay (ELISA). Then, the cells were collected, subjected to PBS washing, and stained with APC-conjugated anti-mouse CD86 antibody and FITC-conjugated anti-mouse CD80 antibody. The expression levels of CD86 and CD80 on the surface of BMDCs were determined by flow cytometry.

### Migration of carriers to LNs

Mice were treated with PBS, OVA/MSN-NH_2_, OVA/MSN-NH-DPM, PVP-MOM, and CTS-MOM. The dose of OVA in each group was 30 µg (OVA-labeled FITC). After 24 h, the draining LNs were collected and processed into a cell suspension in cold PBS. This suspension was then filtered through a 70 µM cell strainer to obtain single-cell suspensions, which were subsequently cultured in 12-well plates 38. The cells were stained with PE-conjugated anti-mouse CD11c antibodies and APC-conjugated anti-mouse CD207 antibodies, followed by analysis by flow cytometry.

### Cellular immune response

Mice were treated with PBS, OVA/MSN-NH_2_, OVA/MSN-NH-DPM, PVP-MOM, and CTS-MOM. All treatments, except for MOM, were administered via subcutaneous injection. The dose of OVA in each group was 30 µg. After 7 days, a boost vaccination was performed. After 14 days of vaccination, spleens were harvested to prepare a cell suspension in cold PBS. Red blood cells within the suspension were subsequently lysed using a red blood cell lysis buffer, and the remaining cells were cultured in 12-well plates. Then, the cells were stimulated using 1 μg/mL SIINFEKL for 6 h, followed by the addition of 2 µg/mL monensin during the final 5 h. After washing with PBS, the cells were stained with FITC-conjugated anti-mouse CD8 antibodies. The cells were then subjected to fixation and permeabilization, followed by intracellular staining with APC-conjugated anti-mouse IFN-γ antibody. Subsequent analysis was performed by flow cytometry.

On the 14th day postvaccination, the mice were euthanized, and the draining LNs were excised to prepare a cell suspension in cold PBS. The suspension was filtered through a 70-µM cell strainer to produce single-cell suspensions, which were then cultured in 12-well plates. The cells were then stained with monoclonal antibodies against CD11c, CD80, CD86, and 25d1.16, and subsequent analysis was conducted by flow cytometry.

### CTL activity

On the 14th day postvaccination, mice were administered an intravenous injection of 1 × 10^7^ CFSE-labeled target cells. These target cells consisted of 50% SIINFEKL-pulsed splenocytes (“CFSE_high_”) labeled with 4 × 10^-6^ mol 5-(and 6)-carboxyfluorescein diacetate succinimidyl ester (CFSE, Invitrogen) and 50% unpulsed splenocytes (“CFSE_low_”) labeled with 0.4 × 10^-6^ mol CFSE. At 18 h postadoptive transfer, the splenocytes were isolated from mice for flow cytometric analysis. The percentage of specific lysis was determined using the following formula 39:

Specific lysis (%) = (1 - (CFSE_low_/CFSE_high_)_naive_ ÷ (CFSE_low_/CFSE_high_)_immunized_) × 100%

where (CFSE_low_/CFSE_high_)_naive_ is the ratio of CFSE_low_-labeled cells to CFSE_high_-labeled cells in naive mice, and (CFSE_low_/CFSE_high_)_immunized_ is that in immunized mice.

### Tumor challenge

For the therapeutic study, the mice were randomly divided into six groups (n = 5). After the subcutaneous injection of 5 × 10^5^ B16-OVA cells, the tumor growth was monitored until 23 days after tumor inoculation. Then, the tumor volumes were determined using the formula V (mm^3^) = (length × width^2^)/2, wherein the length and width were measured using a caliper. Then, the tumors were harvested to prepare a cell suspension in cold PBS. Red blood cells within the suspension were subsequently lysed using a red blood cell lysis buffer, and the remaining cells were cultured in 12-well plates. After washing with PBS, the cells were stained with APC-conjugated anti-mouse CD3 antibodies, PE-conjugated anti-mouse CD4 antibodies, and FITC-conjugated anti-mouse CD8 antibodies. Subsequent analysis was performed by flow cytometry. Another batch of mice was treated in the same method, and their survival rate was evaluated until 40 days after inoculation with B16-OVA cells (n = 6).

### Prevention of tumor growth

For the prevention study, the mice were randomly divided into six groups (n = 5). After two vaccinations administered at 7-day intervals, the mice were inoculated with 5 × 10^5^ B16-OVA cells. Then, the tumor growth was monitored until 23 days after tumor inoculation. The volumes of tumors were determined using the formula V (mm^3^) = (length × width^2^)/2, wherein the length and width were measured using a caliper.

On the 7th day after the 2nd vaccination, the mice were euthanized, and splenocytes were isolated and cultured in 12-well plates, as described previously. The cells were then stained with FITC-conjugated anti-mouse CD8 antibodies, PE-conjugated anti-mouse CD44 antibodies, and APC-conjugated anti-mouse CD62L, followed by analysis by flow cytometry. The supernatant was collected to determine the secretion levels of TNF-α and IL-12 by ELISA.

### Statistical analysis

The error bars depicted in this study represent the standard deviation (SD) derived from a minimum of three independent experiments. Statistical analyses were conducted using SPSS software version 17.0, employing an unpaired two-tailed Student's t-test. Differences between the experimental and control groups were deemed statistically significant at a threshold of p < 0.05. Statistical significance is showed in the figures as follows: *p < 0.05, **p < 0.01, and ***p < 0.001.

## Results and Discussion

### Preparation and characterization of MSN-NH-DPM

**Figure 2:**
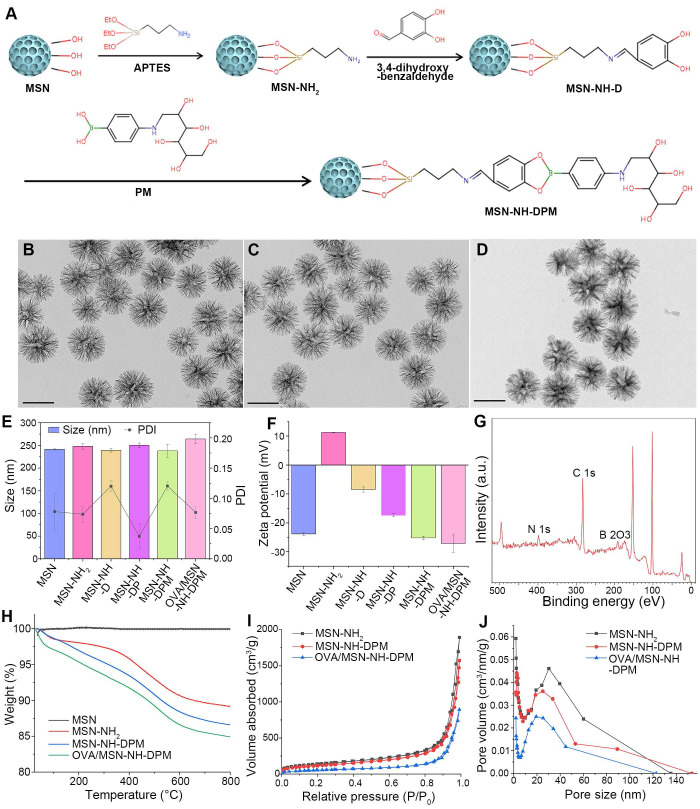
Preparation and characterization of MSN-NH-DPM. (**A**) Graphical synthetic route of MSN-NH-DPM, TEM images of (**B**) MSN-NH_2_, (**C**) MSN-NH-DPM, and (**D**) OVA/MSN-NH-DPM. Scale bar, 200 nm. (**E**) Particle size and corresponding PDI (n = 3). (**F**) Zeta potentials (n = 3). (**G**) X-ray photoelectron spectroscopy analysis of MSN-NH-DPM. (**H**) TGA curve of MSN, MSN-NH_2_, MSN-NH-D, and MSN-NH-DPM. (**I**) Nitrogen adsorption/desorption isotherms and (**J**) corresponding P_D_ curves of MSN-NH_2_, MSN-NH-DPM, and OVA/MSN-NH-DPM. All data are expressed as mean ± SD.

The functional carrier MSN-NH-DPM was constructed for improving intracellular delivery (Figure 2A). To facilitate the loading of OVA within the pores of MSNs and ensure efficient uptake by immune cells, it is essential to tailor the pore size and diameter of MSNs according to the dimensions of the loaded drug and the physiological characteristics of immune cells. OVA has a diameter of approximately 3 nm 40. Concurrently, nanoparticles with a diameter of 200 nm are more readily internalized by immune cells 41. Therefore, the pore size of MSNs must be significantly >3 nm, and their diameter should be ~200 nm. As depicted in Figure 2B, MSN-NH_2_ has an average diameter of 240 nm and exhibited an exceptionally large mesoporous core cone structure. Subsequent vicinal dihydroxylation of MSN-NH_2_ resulted in the formation of MSN-NH-D. Then, the MSN-NH-DPM, which incorporates phenylboronate ester and mannose chemical structures, was synthesized by covalently attaching PM to the dihydroxy groups on the surface of MSN-NH-D. The TEM images of MSN-NH_2_, MSN-NH-DPM, and OVA/MSN-NH-DPM showed that the morphological structure of MSN-NH_2_ remained unchanged after modification with 3,4-dihydroxybenzaldehyde and PM (Figure 2B-D).

Various analytical techniques were used to further characterize the nanoparticles. The hydrodynamic diameter of MSN-NH-DPM was measured as 238 ± 14 nm, and its favorable polydispersity index (PDI) indicates good nanoparticle dispersity (Figure 2E and Table S1). The variations in zeta potential among the different nanoparticles are depicted in Figure 2F. After the amination and vicinal dihydroxylation of MSNs, the zeta potential initially shifted from -23.9 to 11.2 mV (MSN-NH_2_) and then reverted to -8.5 mV (MSN-NH-D). Subsequent PM modification and OVA loading further altered the zeta potential, reducing it from -8.5 to -25.2 mV (MSN-NH-DPM) and further down to -27.5 mV (OVA/MSN-NH-DPM). These changes indicate the successful preparation of MSN-NH-DPM and the effective loading of OVA. We also conducted X-ray photoelectron spectroscopy to characterize the surface elemental composition of MSN-NH-DPM, which revealed a peak corresponding to B 2O3 that confirmed the covalent attachment of PM on the carrier surface (Figure 2G). Furthermore, the TEM images and X-ray photoelectron spectroscopy of MSN-NH-DP revealed that the morphological structure of phenylboronate ester formed by catechol and borate remained unchanged (Figure S1). The mass spectra of DP, DPM, and PM are shown in Figures S2, S3, and S4, respectively. TGA was conducted to determine the grafting quantities at each stage. The grafting percentages for amino, 3,4-dihydroxybenzaldehyde, and PM were determined as 9.35%, 2.26%, and 1.80%, respectively (Figure 2H). Moreover, a nitrogen adsorption-desorption analysis was performed to determine the structural parameters of the nanoparticles (Table S2). The S_BET_ and P_D_ of MSN-NH-DPM were calculated as 520.7 m^2^/g and 25.0 nm, respectively, indicating that there exists ample space for substantial loading of OVA. Additionally, the average LE of OVA in MSN-NH-DPM measured using the Bradford protein assay was 41.1%, and the S_BET_, V_t_, and P_D_ of the carrier progressively decreased after OVA loading, as illustrated in Figure 2I and J. These results conclusively demonstrate the successful preparation of MSN-NH-DPM.

### Preparation and characterization of MOM patches

**Figure 3:**
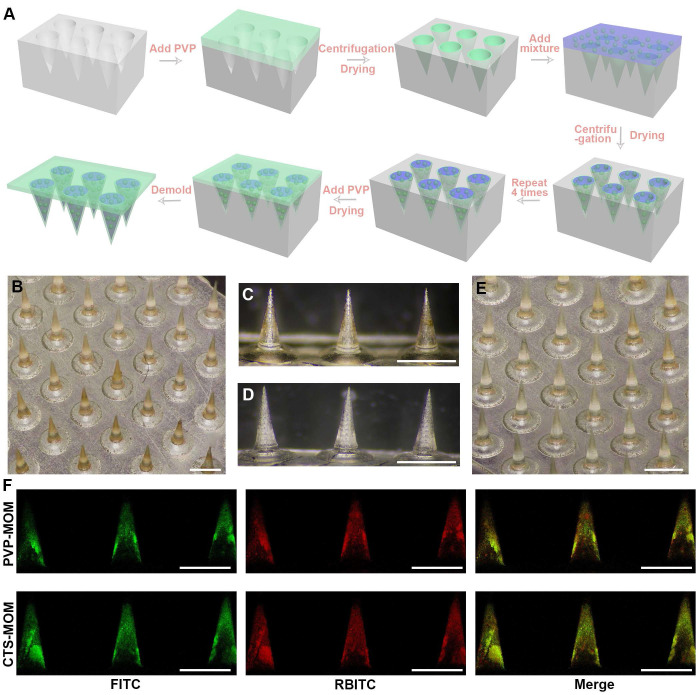
Preparation and characterization of MOM patches. (**A**) Schematic fabrication process of MOM patches. Micrographs of (**B**) CTS-MOM arrays, (**C**) needle tips of CTS-MOM, (**D**) needle tips of PVP-MOM, and (**E**) PVP-MOM arrays. Scale bar, 1000 μm. (F) CLSM images of PVP-MOM and CTS-MOM, where red (RBITC) indicates MSN-NH-DPM, and green (FITC) indicates OVA. Scale bar, 1000 μm.

The manufacturing process of the MN patch is depicted in Figure 3A. Briefly, PVP solution was deposited into the mold by centrifugation and then dried to form a thin shell. Then, a mixture (either PVP solution or a combined solution of PVP and CTS) containing OVA/MSN-NH-DPM was poured into the thin shell to form the main needle. Finally, the PVP solution was added above the needles to obtain a drug-free backing. As shown in Figure 3B-E, the MNs in CTS-MOM and PVP-MOM exhibited a conical morphology, with base diameters of 438 ± 7 and 432 ± 5 μm and heights of 977 ± 6 and 973 ± 7 μm, respectively.

The colocalization of MSN-NH-DPM and OVA was further validated by CLSM (Figure 3F). Due to the repeated centrifugation and the higher density of functionalized MSNs, the nanoparticles tended to aggregate toward the needle tip. Moreover, it was evident that the nanoparticles were almost absent in the backing layer, suggesting that OVA was concentrated in the portion of the needles that penetrated into the skin, thereby minimizing drug waste and ensuring accurate dosage. The OVA/MSN-NH-DPM loadings were examined using the weighing method, as detailed in Table S3. The substantial drug-loading capacity of 278.2 ± 3.61 μg/cm^2^ demonstrates the potential for high-dose transdermal delivery and prolonged therapeutic effects.

### *In vitro* properties of CTS-MOM patches

**Figure 4:**
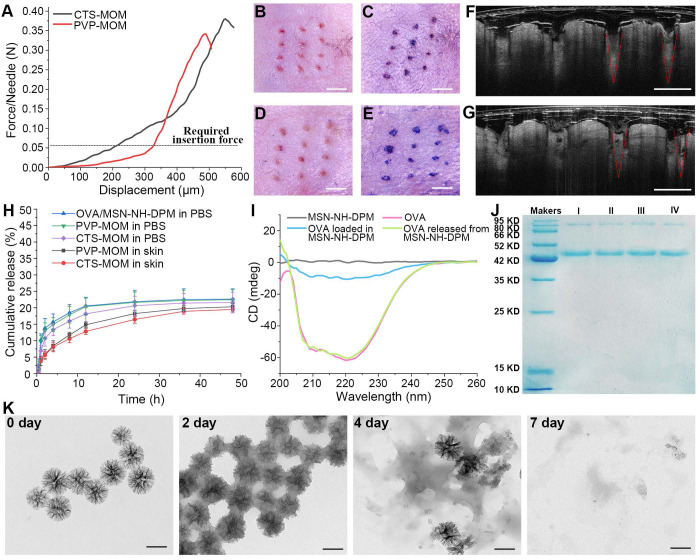
*In vitro* properties of MOM patches. (**A**) Mechanical strength of PVP-MOM and CTS-MOM. Micrographs of the pinholes in the mouse skin after the insertion of (**B**) PVP-MOM and (**D**) CTS-MOM. Scale bar, 1000 μm. Images of the mouse back skin stained with trypan blue after the insertion of (**C**) PVP-MOM and (**E**) CTS-MOM. Scale bar, 1000 μm. OCT images of the insertion of (**F**) PVP-MOM and (**G**) CTS-MOM into the mouse skin. Scale bar, 500 μm. (**H**) *In vitro* release profiles of OVA from OVA/MSN-NH-DPM, PVP-MOM, and CTS-MOM in PBS, and the release profiles of OVA from PVP-MOM and CTS-MOM in the skin. Data represent mean ± SD (n = 3). (**I**) CD spectra of OVA and the OVA loaded into or released from MSN-NH-DPM. (**J**) SDS-PAGE of OVA. (I: OVA, II: OVA released from OVA/MSN-NH-DPM, III: OVA released from PVP-MOM, IV: OVA released from CTS-MOM). (**K**) TEM images of MSN-NH-DPM degraded in simulated body fluids. Scale bars, 200 nm.

For an effective transdermal delivery of the encapsulated drug into systemic circulation, it is imperative for the MNs to penetrate the stratum corneum. Therefore, adequate skin penetration capability and mechanical strength are critical attributes for MNs. The mechanical properties of PVP-MOM and CTS-MOM were investigated using a displacement force apparatus, with the results depicted in Figure 4A. The resistance force showed an increase with greater displacement until a sudden decrease was detected, which was attributed to the bending of the MN tips. Moreover, the minimum failure force recorded for the samples significantly exceeded the force required to breach human skin (0.058 N/needle) 29, indicating that both MNs possessed sufficient mechanical strength to penetrate into the skin without fracturing.

The skin penetration capability of MN patches was comprehensively evaluated using *in vivo* mouse skin models. As depicted in Figure 4B and D, distinct pinholes were observed on the skin, demonstrating that both CTS-MOM and PVP-MOM effectively penetrated the mouse skin. The application of trypan blue, as shown in Figure 4C and E, further corroborated the successful breach of the skin by MOM. To further investigate the penetration of MOM, an OCT microscope was used to capture the real-time images of MOM being inserted into the skin. Figure 4F and G shows the intradermal views of PVP-MOM and CTS-MOM penetrated into the mouse skin. The OCT images provide additional evidence that the MNs can penetrate the skin. Additionally, >80% of MN length was found to successfully penetrate into the mouse skin. Remarkably, the MNs appeared as a relatively brighter region in the OCT images than in the surrounding tissue, potentially due to the shielding effect exerted by the loaded nanoparticles 42. Furthermore, PVP-MOM and CTS-MOM were penetrated into the skin of C57BL/6 mice and then secured with medical tape for 2 h. Then, the mice were sacrificed after a recovery period of 1 day, and the skin at the puncture site of MN was isolated for hematoxylin and eosin staining (Figure S5). Results indicated the absence of significant inflammatory reactions in the mouse skin, suggesting that the MNs demonstrating good biocompatibility.

For the OVA carriers, minimal or slower release of OVA within the skin is preferable, as it allows more antigen to be delivered by the nanoparticles into the cytoplasm of APCs rather than being released extracellularly. The *in vitro* release behaviors of OVA/MSN-NH-DPM, PVP-MOM, and CTS-MOM were determined in PBS. The cumulative release data are illustrated in Figure 4H. OVA exhibited a gradual release from MSN-NH-DPM, with only 20.56% being released within 12 h in PBS. Both PVP-MOM and CTS-MOM could dissolve completely in PBS within 20 min (Figure S6), which minimally affected the release of OVA. Moreover, the mouse skin isolated from C57BL/6 mice was penetrated by PVP-MOM and CTS-MOM to determine the OVA release in transdermal diffusion cell (Figure 4H). Results demonstrated that the release of OVA from PVP-MOM and CTS-MOM was less and slower in the mouse skin than in PBS. It is evident that the slow release of OVA in the skin is advantageous for triggering an effective immune response.

The secondary structures of proteins in vaccines significantly influence their biological functions 43. We analyzed CD spectra to determine whether irreversible alterations would be induced in the secondary structure of OVA due to the adsorption and desorption by the pores of MSN-NH-DPM. The secondary structure of OVA, measured via CD spectra, was altered upon loading into the carrier, probably due to interactions between OVA and MSN-NH-DPM (Figure 4I). However, this alteration was reversed after the release of OVA from MSN-NH-DPM, indicating a temporarily and reversible change in the secondary structure. Moreover, the integrity of the vaccine was determined by SDS-PAGE (Figure 4J). The OVA released from MSN-NH-DPM or CTS-MOM remained almost intact after the MSN modification and drying (Figure S7), indicating that the functionalization of MNs does not compromise the structural integrity of the loaded OVA. To determine the potential for the substantial release of OVA *in vivo*, we examined the degradability of MSN-NH-DPM in simulated body fluids. We observed that MSN-NH-DPM underwent degradation over time, with significant structural breakdown detected in TEM images on the 7th day (Figure 4K). This result suggests that OVA can be effectively released concurrent with the degradation of the carrier. Overall, the CTS-MOM system can function as an effective transdermal vaccine delivery platform, facilitating the transport of vaccines to the sites of immune induction.

### Intracellular delivery and uptake mechanism of OVA/MSN-NH-DPM

**Figure 5:**
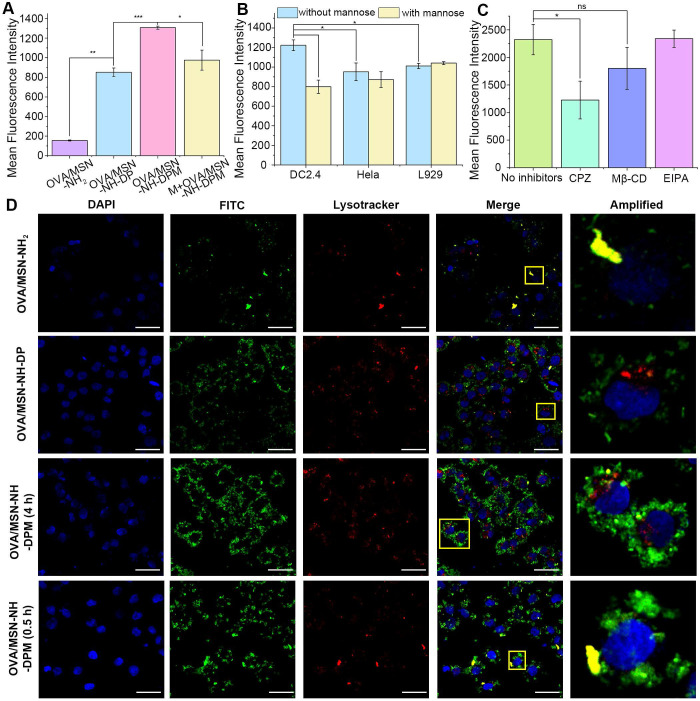
Intracellular delivery of OVA/MSN-NH-DPM. (**A**) *In vitro* cellular uptake of OVA/MSN-NH_2_, OVA/MSN-NH-DP, OVA/MSN-NH-DPM, and M+OVA/MSN-NH-DPM in DC2.4 cells (n = 3). (**B**) Competition assay of OVA/MSN-NH-DPM by adding mannose three cell lines (n = 3). (**C**) Endocytosis pathway analysis of OVA/MSN-NH-DPM in DC2.4 cells (n = 3). (**D**) CLSM images of DC2.4 cells treated with OVA/MSN-NH_2_, OVA/MSN-NH-DP, or OVA/MSN-NH-DPM for 4 h and treated with OVA/MSN-NH-DPM for 0.5 h. The nucleus was stained by DAPI (blue), and the acidic organelles were stained by Lysotracker (red). Scale bars, 20 μm. All data are expressed as mean ± SD. ns, non-significant; *p < 0.05, **p < 0.01, and ***p < 0.001.

The internalization of vaccines by APCs, particularly DCs, is crucial for initiating immune responses 44. The internalization efficiencies of OVA/MSN-NH_2_, OVA/MSN-NH-DP, and OVA/MSN-NH-DPM in DC2.4 cells were investigated by flow cytometry (Figures 5A and S8A). The OVA/MSN-NH-DP group exhibited higher fluorescence intensities than the OVA/MSN-NH_2_ group, suggesting that the presence of phenyl groups improves the internalization of OVA due to their hydrophobic properties. The OVA/MSN-NH-DPM group demonstrated the highest fluorescence intensity, suggesting that mannose, recognized by mannose receptor, improves the uptake by immune cells. Moreover, after pretreatment of DC2.4 cells with mannose for 30 min (M + OVA/MSN-NH-DPM), the uptake of OVA/MSN-NH-DPM was significantly inhibited, showing that the increased cellular uptake was mediated by mannose. To confirm the targeting of MSN-NH-DPM to immune cells, the uptake of OVA/MSN-N-DPM by DC2.4 (immune cells), Hela (lacking mannose receptors 45), and L929 (skin fibroblast) cells was examined by flow cytometry with and without mannose pretreatment (Figures 5B and S8B). Without mannose pretreatment, DC2.4 cells showed significantly higher uptake of OVA/MSN-N-DPM than Hela and L929 cells. After pretreatment with mannose, the uptake of OVA/MSN-N-DPM by DC2.4 cells was significantly inhibited, whereas the uptake efficiency of Hela cells and L929 cells remained unaffected. These findings indicate that mannose enables MSN-N-DPM to exert a certain targeting ability toward immune cells, and after the microneedle release of OVA/MSN-N-DPM into the skin, they are more likely to be taken up by immune cells rather than the abundant skin fibroblasts.

To explore the cellular internalization pathways of OVA/MSN-NH-DPM, various specific inhibitors and Lysotracker Red were used. EIPA, Mβ-CD, and CPZ, which inhibit macropinocytosis-mediated, raft/caveolae-mediated, and clathrin-mediated endocytosis, respectively, were used for identifying the involved endocytosis pathways. In the presence of CPZ and Mβ-CD, the internalization of OVA/MSN-NH-DPM significantly and slightly reduced, respectively, suggesting that clathrin-mediated pathways play a vital role in cellular uptake, whereas raft/caveolae-mediated pathways exert an auxiliary function (Figures 5C and S8C). Previous research has shown that the endocytosis mediated by caveolin bypasses lysosomes and delivers nanoparticles to other organelles, whereas the endocytosis mediated by clathrin can directly deliver nanoparticles to lysosomes 46. Consequently, nanoparticles are more readily disassembled into fragments via clathrin-mediated endocytosis, facilitating the generation of humoral immunity through the MHC-II pathway. We also examined the toxicity of the nanoparticles for DC2.4 cells and observed that the viability of cells treated with MSN-NH-DP and MSN-NH-DPM was >80% even at elevated doses, suggesting the safety of the functionalized MSNs (Figure S9).

We further investigated the endocytosis and intracellular trafficking of protein-vehicle complexes by confocal microscopy. OVA labeled with FITC (OVA-FITC) was effectively internalized into DC2.4 cells via nanoparticle mediation within 4 h (Figure 5D). OVA-FITC was sequestered within lysosomes in the OVA/MSN-NH_2_ group after a 4-h incubation; however, in the OVA/MNS-NH-DP group, it could be clearly observed that OVA-FITC escaped from lysosomes and reached the cytoplasm. Remarkably, the OVA/MSN-NH-DPM group demonstrated significantly higher efficacy in delivering OVA-FITC into DC2.4 cells and promoted more OVA-FITC to escape from lysosomes to the cytoplasm than the OVA/MSN-NH-DP group. DC2.4 cells incubated with OVA/MSN-NH-DPM for 0.5 h exhibited a higher degree of colocalization than those incubated for 4 h (Pearson's coefficient is shown in Table S4). This finding suggests that the nanoparticles initially internalize and reach the endosome before escaping into the cytoplasm. In fact, not all nanoparticles successfully escape; the remaining entrapped in lysosomes are degraded into antigenic peptide fragments, which would probably combine with MHC-II molecules for presentation to CD4 T cells subsequently 47. Conversely, the nanoparticles that escape into the cytoplasm can be presented through the MHC-I pathway, thereby improving cellular immunity.

### Biodistribution and LN accumulation

**Figure 6:**
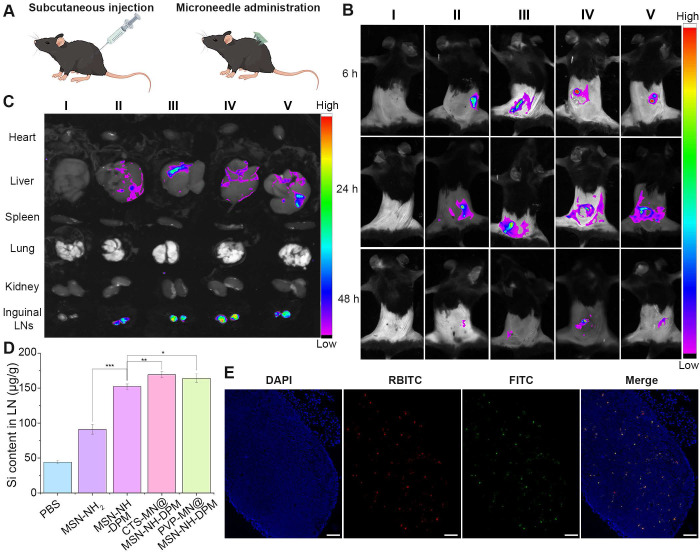
Biodistribution and lymph node accumulation. (**A**) Schematic of vaccination for injection and MN administration. (**B**) Migration of fluorescence-labeled nanoparticles from the administration site to other sites *in vivo*. (I: PBS, II: MSN-NH_2_, III: MSN-NH-DPM, IV: CTS-MN@MSN-NH-DPM, V: PVP-MN@MSN-NH-DPM). (**C**) Distribution of nanoparticles in various organs. (I: PBS, II: MSN-NH_2_, III: MSN-NH-DPM, IV: CTS-MN@MSN-NH-DPM, V: PVP-MN@MSN-NH-DPM). (**D**) Si content in inguinal LNs (n = 3). Data are means ± SD (n = 3). (**E**) Colocalization images of OVA and MSN-NH-DPM in inguinal LN tissue sections after vaccination with CTS-MOM. Scale bars, 100 μm. *p < 0.05, **p < 0.01, and ***p < 0.001.

The migration of antigen to LNs is essential for the effective initiation of interactions between DCs and T cells, subsequently resulting in the antigen-specific CTL response against tumors 48. MSN-NH_2_ and MSN-NH-DPM were injected into the flank, and CTS-MN@MSN-NH-DPM and PVP-MN@MSN-NH-DPM were vaccinated at the same site in C57BL/6 mice (Figure 6A). The mice were then examined using an *in vivo* imaging system (IVIS). The group treated with microneedles had more nanoparticles remaining at the administration site than that observed with subcutaneous injection at 6 h, which is because of the lower water content in the skin than in the subcutaneous layer. In the three groups of MSN-NH-DPM, CTS-MN@MSN-NH-DPM, and PVP-MN@MSN-NH-DPM, it could be clearly observed that the nanoparticles migrated from the administration site to inguinal LNs in 24 h, indicating that DPM could promote the uptake of nanoparticles by immune cells and carry them to LNs (Figure 6B). Due to the low limit of detection of *in vivo* fluorescence signals, various organs were isolated for IVIS imaging 24 h postvaccination (Figure 6C). Further analysis of the isolated organs revealed that the nanoparticles predominantly accumulated in the liver and inguinal LNs. Remarkably, mannose-modified nanoparticles exhibited a higher accumulation in LNs than unmodified nanoparticles. The accumulation of LNs after vaccination with MSN-NH-DPM and PVP-MN@MSN-NH-DPM was approximately equivalent. Nevertheless, vaccination with CTS-MN@MSN-NH-DPM resulted in a greater accumulation. This result suggests that MN vaccination can achieve comparable effects to those of subcutaneous injection, whereas chitosan may increase DC activity, facilitating the transfer of nanoparticles to LNs.

Quantitative analysis of silicon (Si) contained within MSNs in inguinal LNs was conducted by ICP-MS to confirm the accumulation of nanoparticles in LNs. Figure 6D shows the Si content in inguinal LNs 24 h after the subcutaneous injection of MSN-NH_2_ and MSN-NH-DPM or vaccination with PVP-MN@MSN-NH-DPM and CTS-MN@MSN-NH-DPM. Results showed a higher Si content in the MSN-NH_2_ group than in the control group (PBS), suggesting that MSN-NH_2_ can be drained into LNs because of their small size. The Si content in the MSN-NH-DPM group was significantly higher than that in the MSN-NH_2_ group, indicating that MSN-NH-DPM also promotes immune cell uptake via its mannose structure, thereby being carried to LNs. Furthermore, the Si content in the PVP-MN@MSN-NH-DPM group was slightly higher than that in the MSN-NH-DPM group, suggesting that the high concentration of immune cells in the skin provides some assistance. The highest Si content was detected in the CTS-MN@MSN-NH-DPM group, emphasizing the role of chitosan in activating immune cells. We further examined the colocalization of OVA and its carrier within inguinal LNs (Figures 6E and S10). Remarkably, the red fluorescence emitted by RBITC-MSN-NH-DPM exhibited a substantial overlap with the green fluorescence of FITC-OVA in the CTS-MOM group, suggesting that OVA can be effectively transported to inguinal LNs with MSN-NH-DPM. These results collectively suggest that CTS-MOM facilitates the accumulation of vaccines within LNs.

### Ability to induce immune responses* in vitro*

**Figure 7:**
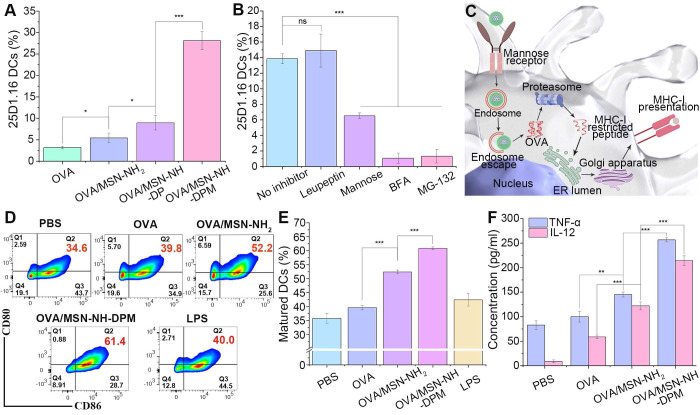
Ability to induce immune responses* in vitro*. (**A**) Cross-presentation of OVA through different nanocarriers (n = 3). (**B**) Cross-presentation pathway analysis of OVA/MSN-NH-DPM by several inhibitors in DC2.4 cells (n = 3). (**C**) Schematic of the promotion of OVA cross-presentation by MSN-NH-DPM through the “cytosolic” pathway in DC2.4 cells. (**D**) Representative cytometry plots of BMDCs expressing CD80 and CD86 treated with nanoparticles. (**E**) Percentages of matured dendritic cells that simultaneously express CD80 and CD86 (n = 3). (**F**) Secretion of TNF-α and IL-12 measured by ELISA (n = 3). All data are expressed as mean ± SD. ns, non-significant; *p < 0.05, **p < 0.01, and ***p < 0.001.

It has been confirmed that OVA/MSN-NH-DPM can reach the cytoplasm through endosomal escape. Hence, we next investigated the cross-presentation of antigens by flow cytometry by detecting H-2Kb-SIINFEKL complexes on the surface of DC2.4 cells labeled with the monoclonal antibody 25d1.16. Results showed that OVA/MSN-NH_2_, OVA/MSN-NH-DP, and OVA/MSN-NH-DPM increased the proportion of 25d1.16-positive cells by 1.7-, 2.8-, and 8.8-fold, respectively, compared with free OVA (Figure 7A). These findings collectively indicate that DP improved the ability of cross-presentation through endosomal escape, and mannose further strengthened the cross-presentation by increasing cellular uptake. We then used mannose, BFA, MG-132, and leupeptin 49, which inhibit mannose receptor, protein transport from the endoplasmic reticulum to Golgi apparatus, proteasome, and lysosomal protease, respectively, to identify the cross-presentation pathway involved in OVA/MSN-NH-DPM (Figure 7B). Mannose reduced the proportion of 25d1.16-positive cells. Pretreatment of DC2.4 cells with BFA or MG-132 instead of leupeptin significantly decreased the proportion of 25d1.16-positive cells. These findings indicate that OVA/MSN-NH-DPM was first taken up by cells through the mannose receptor, and then via the cytosolic rather than lysosomal pathway, driving its cross-presentation in DC2.4 cells (Figure 7C).

Promoting DCs to maturation is critical for generating antigen-specific immune responses after vaccination 50. To determine the ability to promote BMDC maturation, we measured the expression levels of CD80 and CD86 (markers of BMDC maturation) after 24 h of coculturing with OVA, OVA/MSN-NH_2_, or OVA/MSN-NH-DPM. BMDCs treated with lipopolysaccharide and PBS served as positive and negative controls, respectively. The cell flow analysis strategy is depicted in Figure S11. As illustrated in Figure 7D and E, the OVA/MSN-NH_2_ group showed significantly increased coexpression of CD80 and CD86 on BMDCs compared with the OVA group, indicating the potential of MSN as an adjuvant. Simultaneously, the OVA/MSN-NH-DPM group showed significantly increased expression of CD80 and CD86 compared with the OVA and OVA/MSN-NH_2_ groups, indicating that an efficient intracellular delivery of nanoparticles is beneficial for DC maturation.

A further indication of DC maturation is the secretion of proinflammatory cytokines. After maturation, DCs exhibit increased secretion of interleukin 12 (IL-12) and tumor necrosis factor alpha (TNF-α). IL-12 primarily induces type I helper T cells (Th1), and TNF-α is pivotal in the antitumor immune response; the increased secretion of these two factors contributes to cancer therapy. We quantified the levels of TNF-α and IL-12 secreted by BMDCs incubated with various samples by ELISA (Figure 7F). Remarkably, OVA loaded into MSN-NH_2_ significantly augmented the secretion of cytokines, suggesting an adjuvant effect of the carrier. Moreover, the cytokine concentration in the OVA/MSN-NH-DPM group was almost double that in the OVA/MSN-NH_2_ group, suggesting that the combination of intracellular delivery and improved uptake triggers more robust immune responses.

### Ability to induce immune responses* in vivo*

**Figure 8:**
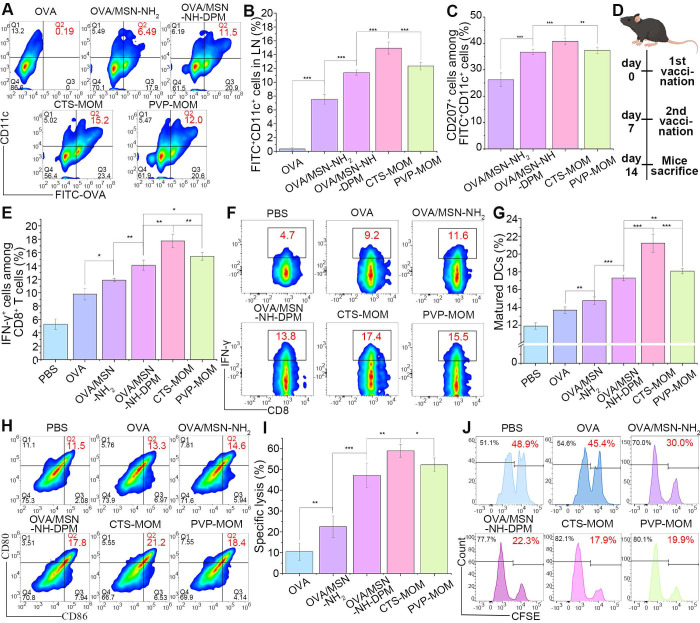
Ability to induce immune responses* in vivo.*** (A)** Representative cytometry plots of FITC^+^ on DCs. **(B)** Percentages of FITC^+^ on DCs (n = 5). **(C)** Percentages of CD207^+^ on FITC^+^ CD11c^+^ cells (n = 5). **(D)** Schematic timeline of vaccination for mice. **(E)** Percentages of IFN-γ^+^ on CD8^+^ T cells (n = 5). (**F**) Representative cytometry plots of IFN-γ^+^ on CD8^+^ T cells. (**G**) Percentages of matured DCs (n = 5). (**H**) Representative flow cytometry plots of CD80^+^ CD86^+^ on DCs. (**I**) Percentage of specific lysis induced by CTL response (n = 5). (**J**) Representative flow cytometry plots of CFSE_high_-labeled and CFSE_low_-labeled splenocytes. All data are expressed as mean ± SD. *p < 0.05, **p < 0.01, and ***p < 0.001.

The migration of antigens to LNs is essential for the effective initiation of interactions between DCs and T cells, ultimately resulting in an antigen-specific CTL response against tumors. We injected mice with OVA, OVA/MSN-NH_2_, and OVA/MSN-NH-DPM and treated them with PVP-MOM and CTS-MOM (OVA-labeled FITC). After 24 h, the draining LNs were harvested and homogenized, and the cells were isolated for culture and restimulation. The cells were then incubated with PE-conjugated anti-mouse CD11c antibodies and APC-conjugated anti-mouse CD207 antibodies, followed by analysis by flow cytometry (Figure S12). FITC^+^ PE^+^ cells were the DCs uptaking OVA (Figure 8A and B). Results showed that it was difficult for free OVA to migrate from the injection site to the draining LNs. In contrast, the increased presence of OVA/MSN-NH_2_ suggested that the carrier exerted an adjuvant effect. The highest proportion of DCs was detected in the CTS-MOM group, which was attributed to the combined action of the various structural components. Moreover, FITC^+^ PE^+^ APC^+^ cells were identified as Langerhans cells (LCs), a subset of skin DCs that migrate to the draining LNs after internalizing the sample (Figures 8C and S13). Remarkably, there was a 1.4-fold increase in OVA/MSN-NH-DPM compared with that in OVA/MSN-NH_2_, indicating a greater number of DCs carrying OVA from the skin.

IFN-γ can inhibit tumor progression by suppressing cancer cells or inducing the regression of tumor vasculature 51. The presence of CD8^+^ T cells indicates the induction of cellular immunity, with IFN-γ serving as a pivotal effector molecule in tumor rejection. Therefore, we collected spleens from the immunized mice to evaluate CD8^+^ T cells secreting IFN-γ. Mice were subcutaneously injected with OVA, OVA/MSN-NH_2_, and OVA/MSN-NH-DPM and treated with PVP-MOM and CTS-MOM on day 7 (prime) and day 14 (boost) (Figure 8D). Subsequent to harvesting, the spleens were homogenized, and the cells were isolated for culture and restimulation. The proportions of IFN-γ-producing CD8^+^ T cells were then detected (Figure 8E and F). The OVA/MSN-NH_2_ group provoked a robust cytotoxic CD8^+^ T-cell response compared with the OVA group, demonstrating the adjuvant effects of MSNs. Moreover, the significantly increased response detected in the OVA/MSN-NH-DPM group suggests that the improved uptake and endosomal escape could facilitate cellular immunity. In particular, PVP-MOM resulted in a higher proportion of IFN-γ-producing CD8^+^ T cells than OVA/MSN-NH-DPM, indicating that MN vaccination can improve immune responses from the abundant immune cells in the skin. Additionally, the increased production of IFN-γ triggered by CTS-MOM suggests that chitosan serves as an effective vector for antitumor vaccines. After the vaccination of mice with various samples, the draining LNs were homogenized, and the cells were isolated for culture and restimulation. Subsequently, the proportions of 25d1.16-, CD80-, and CD86-positive DCs were measured (Figures 8G, H and S14). A similar trend to that of IFN-γ level was observed, wherein CTS-MOM exhibited the most significant effect in improving the immune response.

These results together indicate that the system has the potential to induce an effector phenotype in CD8^+^ T cells; however, it still remains unascertained whether this induction is sufficient for immune cells to effectively recognize and eliminate antigen-bearing target cells. To explore this issue, we determined whether the established vaccine delivery system could stimulate CTL activity (Figure 8I and j). Specifically, SIINFEKL-stimulated CFSE_high_-labeled splenocytes and CFSE_low_-labeled splenocytes were mixed in a 1:1 ratio and then injected into mice that had been vaccinated as described previously. Examination of OVA-specific lysis on day 14 revealed a 4.5-fold increase in target cell lysis in the OVA/MSN-NH-DPM group compared with that in the OVA-alone group and a 2.1-fold increase compared with that in the OVA/MSN-NH_2_ group. The most remarkable lysis was detected in the CTS-MOM group, indicating that the CTS-MOM vaccine is more effective in triggering an antigen-specific cellular immune response against cancer.

### Anticancer efficiency of CTS-MOM

**Figure 9:**
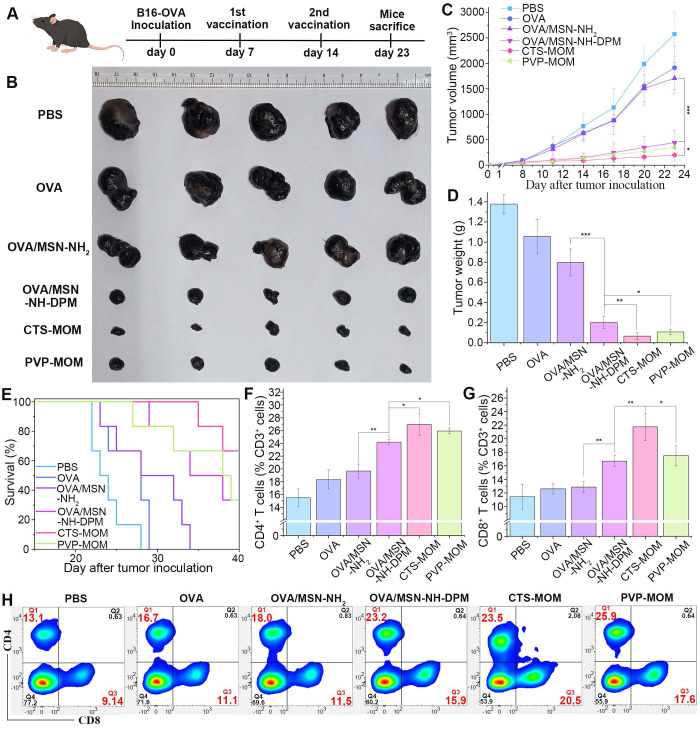
Anticancer efficiency of CTS-MOM in the melanoma mouse model. (**A**) Schematic timeline of vaccination against tumor. (**B**) Representative images of tumors harvested on the 23rd day. (**C**) Tumor volume and (**D**) tumor weight until day 23 after tumor inoculation (n = 5). (**E**) Survival rate until day 40 after tumor inoculation (n = 6). Proportion of tumor-infiltrating (**F**) CD4^+^ T cells and (**G**) CD8^+^ T cells in cells harvested from tumor tissues (n = 5). (**H**) Representative cytometry plots of CD4^+^ and CD8^+^ T cells (gated on CD3^+^) in treated tumors. All data are expressed as mean ± SD. *p < 0.05, **p < 0.01, and ***p < 0.001.

To further determine the potential application of CTS-MOM as a cancer vaccine, we conducted an *in vivo* study to explore its tumor suppression capability in a mouse model. Female C57BL/6 mice, aged 7 weeks, were subcutaneously injected with 5 × 10^5^ B16-OVA cells, and the tumors were allowed to develop over 7 days to reach a size that is visible to the naked eye. Then, the mice received subcutaneous injections of PBS, OVA, OVA/MSN-NH_2_, and OVA/MSN-NH-DPM and vaccination with PVP-MOM and CTS-MOM, with a 7-day interval between the two administrations (Figure 9A). Tumors were harvested on the 23rd day for photography (Figure 9B), monitoring the tumor size (Figure 9C and Table S5), and weighing (Figure 9D). Compared with the control group (PBS), both the OVA and OVA/MSN-NH_2_ groups exhibited modest suppression of tumor growth and survival rates. In contrast, the OVA/MSN-NH-DPM vaccine group showed a significant reduction in tumor growth, which is attributed to its increased cellular uptake and intracellular delivery. Furthermore, compared with OVA/MSN-NH-DPM, the suppression of PVP-MOM on tumors slightly increased, and that of CTS-MOM was greater. Another batch of mice was selected for the same administration procedure, and the survival status within 40 days was recorded (Figure 9E). Consistent with the abovementioned results, CTS-MOM demonstrated the highest survival rate within 40 days after tumor inoculation. Altogether, these results strongly suggest that CTS-MOM holds potential as a platform for improving the antitumor immune responses of vaccines.

To identify infiltrated cytotoxic T cells within tumors, treated tumors were collected, and tumor-infiltrating lymphocytes (TILs) were examined by flow cytometry. After isolating the cells from the tumor tissue, CD3^+^ (marker of T lymphocytes), CD4^+^ (marker of helper T cells), and CD8^+^ (marker of cytotoxic T cells) were used for identifying TILs (Figures 9H and S15). The proportions of CD4^+^ helper T cells significantly increased in tumors treated with OVA/MSN-NH-DPM, and vaccination with PVP-MOM and CTS-MOM was more effective than subcutaneous injection (Figure 9F). Moreover, tumors treated with OVA/MSN-NH-DPM were significantly infiltrated by CD8^+^ T cells compared with tumors treated with OVA/MSN-NH_2_ (Figure 9G). The proportion of CD8^+^ T cells in the CTS-MOM group was 21.7% ± 2.0%, which was almost twice that in the OVA/MSN-NH_2_ group (12.9% ± 0.9%). Therefore, we confirmed that the proposed antigen intracellular delivery system can induce a large number of helper T cells and cytotoxic T cells to infiltrate into tumors and activate immune responses.

### Preventive efficacy of CTS-MOM

**Figure 10:**
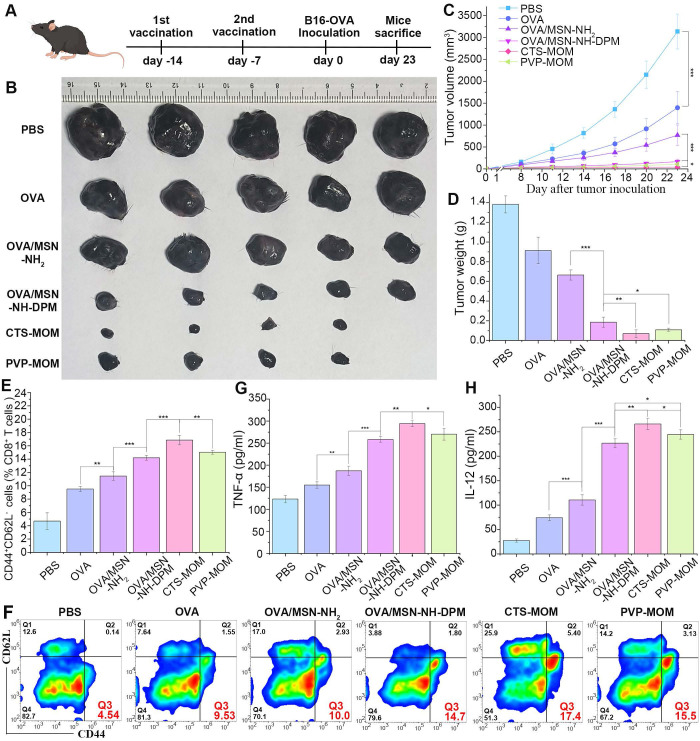
Preventive efficacy of CTS-MOM in a melanoma mouse model. (**A**) Schematic timeline of vaccination for preventing tumors. (**B**) Representative images of tumors harvested on the 23rd day. (**C**) Tumor volume and (**D**) tumor weight until day 23 after tumor inoculation (n = 5). (**E**) Percentages of CD44^+^ CD62L^+^ on CD8^+^ T cells (n = 5). (**F**) Representative flow cytometry plots of CD44^+^ CD62L^+^ on CD8 T^+^ cells. Secretion of (**G**) TNF-α and (**H**) IL-12 in mice measured by ELISA (n = 5). All data are expressed as mean ± SD. *p < 0.05, **p < 0.01, and ***p < 0.001.

In the context of tumor vaccines, preventive efficacy serves as a crucial criterion for evaluation. Female C57BL/6 mice were vaccinated with different samples, with a 7-day interval between two administrations. The mice were then subcutaneously injected with 5 × 10^5^ B16-OVA cells, and the tumors were allowed to develop for 7 days until they became visible to the naked eye (Figure 10A). The tumors were harvested on the 23rd day for photography (Figure 10B), monitoring tumor size (Figure 10C and Table S6), and weighing (Figure 10D). Results demonstrated that OVA exerted a significant tumor-inhibiting effect compared with that in the control group, suggesting that prevention plays a more vital role in antitumor strategies than treatment. Moreover, CTS-MOM exerted the most remarkable antitumor effect overall, indicating its potential utility as a tumor vaccine.

Central memory T cells primarily reside in secondary lymphoid organs to mediate the proliferation and differentiation of immune cells, and effector memory T cells (T_CM_) exhibit the ability to rapidly migrate to response sites and induce immune responses. To further explore the potential of CTS-MOM in exerting an immune memory effect, we examined T_CM_ (CD44^+^CD62L^-^) from spleens to evaluate the generation of memory immunity on the 7th day after the second vaccination with different samples (Figure 10E and F). Results indicated that OVA/MSN-NH-DPM significantly improved the induction of CD8^+^ T_CM_ compared with OVA/MSN-NH_2_, and CTS-MOM exerted the most remarkable effect in promoting immune memory. We also measured the levels of TNF-α and IL-12 secreted by splenocytes by ELISA (Figure 10G and H). We detected a 1.38-fold increased secretion of TNF-α and a 2.05-fold increased secretion of IL-12 in the OVA/MSN-NH-DPM group compared with those in the OVA/MSN-NH_2_ group, and CTS-MOM induced the highest levels of the two inflammatory factors. These data suggest that CTS-MOM possesses the capacity to trigger a more robust memory immune response, potentially contributing to cancer prevention.

## Conclusions

We propose an innovative cancer vaccine delivery system that increases the intracellular delivery efficiency of phenylboronate ester through chitosan MNs and mannose. The customized MSN-NH_2_ features an enlarged pore diameter, thereby facilitating high-capacity storage. The phenylboronate ester structure enabled the nanoparticles to reach the cytoplasm through endosomal escape, thus augmenting cellular immune responses. The mannose component in MSN-NH-DPM improved the uptake efficiency of nanoparticles by immune cells, facilitating their entry into and escape from endosomes to the cytoplasm. Furthermore, chitosan MNs improved the immunogenic effect of OVA compared with that exerted by subcutaneous injection. Moreover, the administration of OVA/MSN-NH-DPM via chitosan MNs to the skin successfully delivered nanocarriers to nearby LNs, triggering a robust cellular immune response, effectively inhibiting tumor progression, and extending the survival time. Additionally, considering that the targets of DNA and mRNA vaccines are also located in the cytoplasm of immune cells, this delivery strategy may be applicable to nucleic acid vaccines.

## Supplementary Material

Supplementary materials and methods, figures and table.

## Abbreviations

MSN: mesoporous silica nanoparticles
MNs: microneedle patches
MHC: major histocompatibility complex
DCs: dendritic cells
LNs: lymph nodes
APCs: antigen-presenting cells
OVA: Ovalbumin
MOM: MNs containing OVA/MSN-NH-DPM
PVP: polyvinylpyrrolidone
CTS: chitosan
CTLs: cytotoxic T lymphocytes
GM-CSF: Murine granulocyte-macrophage colony-stimulating factor
PDMS: Female polydimethylsiloxane
LE: loading efficiency
TEM: transmission electron microscope
CTS-MOM: CTS-MN containing OVA/MSN-NH-DPM
PVP-MOM: PVP-MN containing OVA/MSN-NH-DPM
PBS: phosphate-buffered saline
CD: circular dichroism
SDS-PAGE: sodium dodecyl sulfate polyacrylamide gel electrophoresis
OCT: optical coherence tomography
EIPA: amiloride hydrochloride
Mβ-CD: methyl-β-cyclodextrin
CPZ: Chlorpromazine
CLSM: confocal laser scanning microscopy
BMDCs: Bone marrow-derived dendritic cells
CFSE: 5-(and 6)-carboxyfluorescein diacetate succinimidyl ester
