# Supplementary material for: Mannose and phenylboronate ester functionalized mesoporous silica nanoparticles contained in chitosan microneedles for enhancing cellular immunity and antitumor efficacy
Source: Theranostics. 2026 Jan 1;16(4):2080–100. doi: 10.7150/thno.121610 (PMC12680897; doi:10.7150/thno.121610)
Supplement: Supplementary file 1 — Supplementary materials and methods, figures and table. [file thnov16p2080s1.pdf]

Supplementary Information

# Mannose and Phenylboronate Ester Functionalized Mesoporous Silica Nanoparticles Contained in Chitosan Microneedles for Enhancing Cellular Immunity and Antitumor Efficacy

*Nanxi Chen[a, 1], Ye He[a, 1], Yuxuan Zhang[b], Yong Cui[b], Hongyan Lu[a], Jinghai Zhang[b], Haotian Zhang[c], Qinfu Zhao[a], Yuling Mao[a],, Yikun Gao[b,]* Siling Wang[a,]**

[a] Department of Pharmaceutics, School of Pharmacy, Shenyang Pharmaceutical University, Shenyang 110016, China

[b] Department of Biomedical Engineering, School of Medical Devices, Shenyang Pharmaceutical University, Shenyang 110016, China

[c] Department of Pharmacology, Shenyang Pharmaceutical University, Shenyang 110016, China

[*]Corresponding authors
[1]These authors contributed equally.

E-mails: yikungao@syphu.edu.cn (Y Gao); silingwang@syphu.edu.cn (S Wang).

# Materials and methods

## 1. Preparation of DPM

The synthesis of PM was achieved by conjugating mannose with 4-aminophenylboronic acid through a Schiff base reaction. Subsequently, 3,4-dihydroxybenzaldehyde was esterified with PM to obtain DPM. In brief, 4-aminophenylboronic acid (17.4 mg, 0.1 mmol) and mannose (18.0 mg, 0.1 mmol) were dissolved in water, followed by the addition of sodium triacetoxyborohydride (31.8 mg, 0.15 mmol) to react for 48 h at 25 ℃ to obtain PM. Subsequently, 3,4-dihydroxybenzaldehyde (13.8 mg, 0.1 mmol) was added into the solution, and the pH was adjusted to approximately 9.2 via triethylamine and stirred at 25 ℃ for 12 hours to synthesize DPM.

## 2. Preparation of fluorescently labeled nanoparticles and OVA

RBITC-labeled MSN-$NH_2$ or MSN-NH-DPM were prepared by mixing the nanoparticles with RBITC and stirring at 25 ℃ for 24 hours. The final fluorescently labeled products were obtained through centrifugation and washing. OVA was labeled with FITC via the reaction between the amino groups and isothiocyanate groups, with unincorporated dye removed by ultrafiltration centrifugation.

## 3. Drug loading capacity of CTS-MOM

3.4 mg of OVA/MSN-NH-DPM was incorporated into a 100 mg mixture (PVP and CTS in a mass ratio of 7:3), resulting in a 3.29% concentration of OVA/MSN-NH-DPM in the substrate solution. The loading efficiency of OVA in MSN-NH-DPM was determined to be 41.1%, and the area of the MNs was measured to be 1.69 $cm^2$. The OVA loading capacity of CTS-MOM was calculated using the following equation.

$$\text{loading capacity } (mg/cm^2) = Mixture_{total} \times 3.29\% \times 41.1\% / 1.69$$

Where $Mixture_{total}$ is the weight of the mixture containing OVA/MSN-NH-DPM loaded into the

mold's pinhole after four cycles of addition and drying. And the OVA loading capacity of CTS-MOM was 278.2 ± 3.61 μg/cm$^2$.

## 4. The isolation and culture of BMDCs

BMDCs were isolated from the bone marrow of C57BL/6 mice as previously described [1]. In brief, bone marrow cells were harvested from the femur and tibia of the mice and then red blood cell lysis buffer was added to lyse the red blood cells. Following this, cells cultured at a concentration of $5 \times 10^6$ per 12-well plates with 12 mL of RPMI 1640 medium supplemented with 10% FBS, 1% penicillin-streptomycin, and 20 ng/mL GM-CSF. On day 3, an additional 10 mL of fresh medium was added. Then on day 6, half of the supernatant was replaced with fresh medium. By day 8, nonadherent and loosely adherent cells were harvested to evaluate the purity of the BMDCs. BMDCs exhibiting a CD11c-positive cell percentage exceeding 80% were used.

**Table S1** Polydispersity index (PDI) of MSN, $MSN\text{-}NH_2$, MSN-NH-D, MSN-NH-DPM and OVA/MSN-NH-DPM.

| Sample | PDI |
|---|---|
| MSN | 0.078±0.031 |
| $MSN\text{-}NH_2$ | 0.074±0.013 |
| MSN-NH-D | 0.120±0.009 |
| MSN-NH-DPM | 0.120±0.007 |
| OVA/MSN-NH-DPM | 0.077±0.010 |

**Table S2** Specific surface areas ($S_{BET}$), pore volumes (Vt) and pore diameter ($P_D$) of $MSN\text{-}NH_2$, MSN-NH-DPM and OVA/MSN-NH-DPM.

| Sample | $S_{BET}$ ($m^2/g$) | Vt ($cm^3/g$) | $P_D$ (nm) |
|---|---|---|---|
| $MSN\text{-}NH_2$ | 597.62 | 2.82 | 30.2 |
| MSN-NH-DPM | 520.72 | 2.37 | 25.0 |
| OVA/MSN-NH-DPM | 270.33 | 1.33 | 19.4 |

**Table S3** Weight of the mixture containing OVA/MSN-NH-DPM loading into the pinhole of MN mold after four cycles of addition and drying.

| Batch number of CTS-MM | Weight of $Mixture_{1th}$ (mg) | Weight of $Mixture_{2th}$ (mg) | Weight of $Mixture_{3th}$ (mg) | Weight of $Mixture_{4th}$ (mg) |
|---|---|---|---|---|
| 1 | 12.3 | 10.3 | 7.2 | 4.5 |
| 2 | 13.9 | 10.5 | 6.6 | 3.8 |
| 3 | 14.4 | 8.7 | 7.9 | 4.2 |

**Table S4** Pearsons coefficient of $MSN\text{-}NH_2$ (4 h), MSN-NH-DP (4 h), MSN-NH-DPM (4 h) and MSN-NH-DPM (0.5 h).

| Sample | Pearsons coefficient |
|---|---|
| $MSN\text{-}NH_2$ (4 h) | 0.83 |
| MSN-NH-DP (4 h) | 0.37 |
| MSN-NH-DPM (4 h) | 0.26 |
| MSN-NH-DPM (0.5 h) | 0.58 |

**Table S5** Maximum diameter/volume of tumors for treatment

| Tumors | Maximum diameter (mm) | Volume ($mm^3$) |
|---|---|---|
| PBS | 19.05±0.68 | 2572.88±437.42 |
| OVA | 17.94±2.22 | 1913.79±424.99 |
| OVA/MSN-$NH_2$ | 16.55±1.82 | 1708.25±302.61 |
| OVA/MSN-NH-DPM | 10.40±1.78 | 424.83±235.35 |
| CTS-MOM | 7.94±1.80 | 200.09±120.69 |
| PVP-MOM | 9.49±3.02 | 349.00±203.80 |

**Table S6** Maximum diameter/volume of tumors for prevention

| Tumors | Maximum diameter (mm) | Volume ($mm^3$) |
|---|---|---|
| PBS | 19.16±0.73 | 3134.66±397.91 |
| OVA | 16.33±0.97 | 1396.19±366.46 |
| OVA/MSN-$NH_2$ | 14.59±1.85 | 769.08±230.67 |
| OVA/MSN-NH-DPM | 8.39±1.57 | 166.24±53.64 |
| CTS-MOM | 4.63±0.48 | 40.43±8.33 |
| PVP-MOM | 6.74±0.61 | 106.62±21.43 |

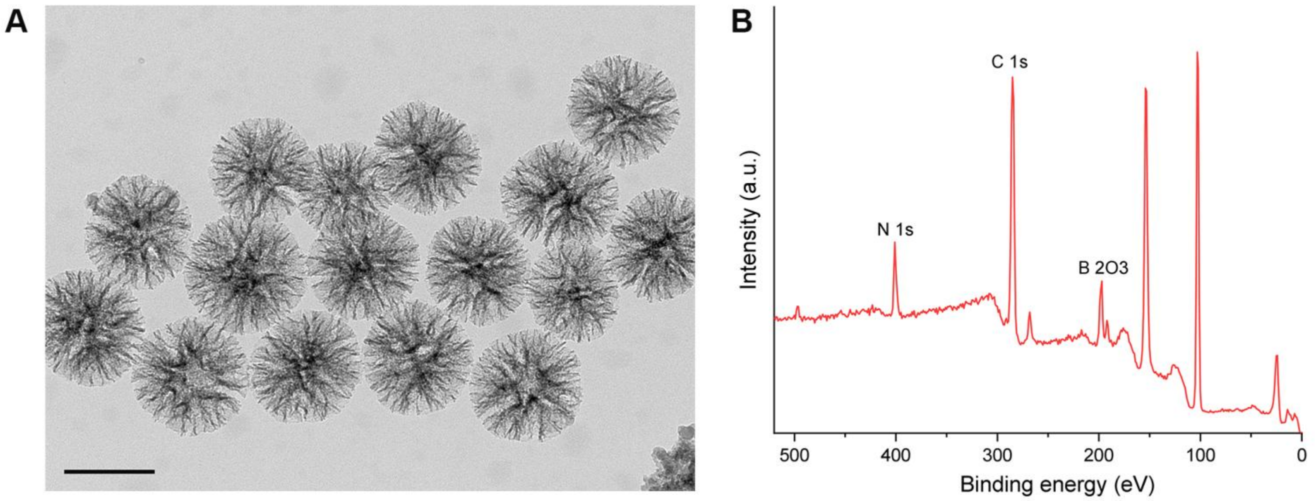


**Figure S1.** (**A**) TEM images of MSN-NH-DP. Scale bar, 200 nm. (**B**) X-ray photoelectron spectroscopy analysis of MSN-NH-DP.

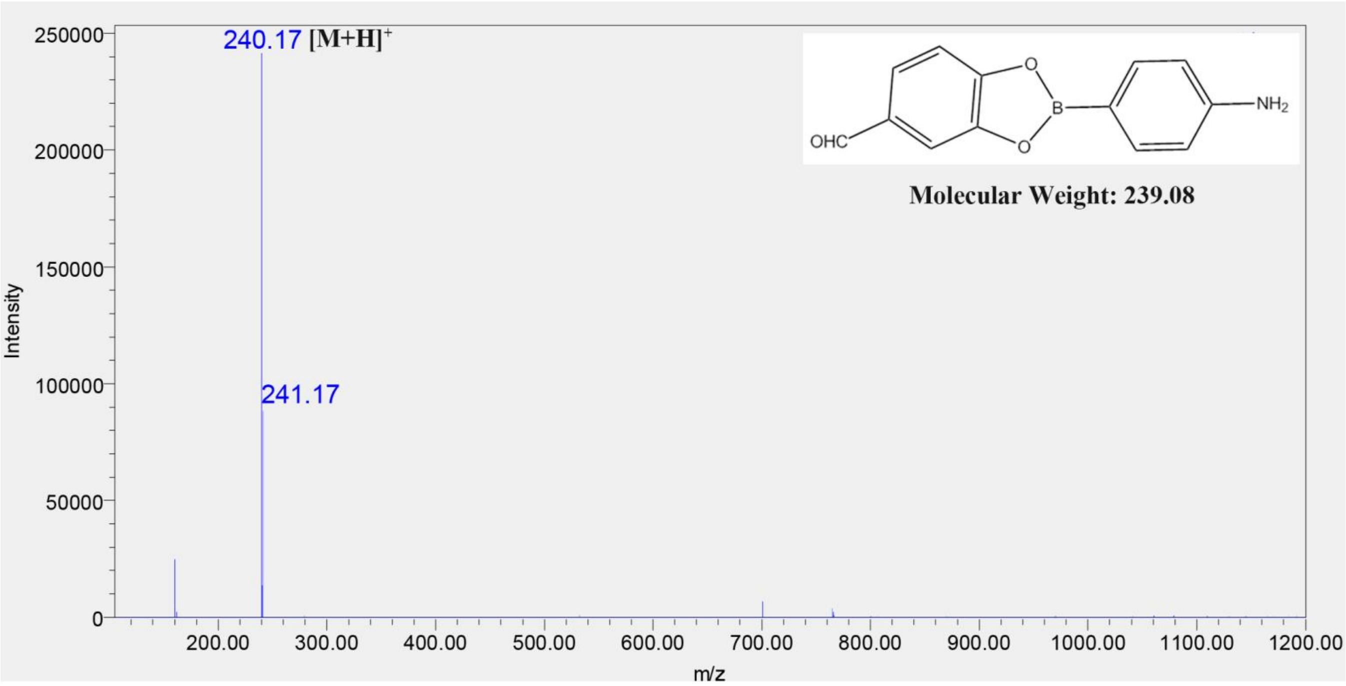


**Figure S2.** Mass spectrum and chemical structure of DP. m/z = 240.17 ([M+H]$^+$) for DP.

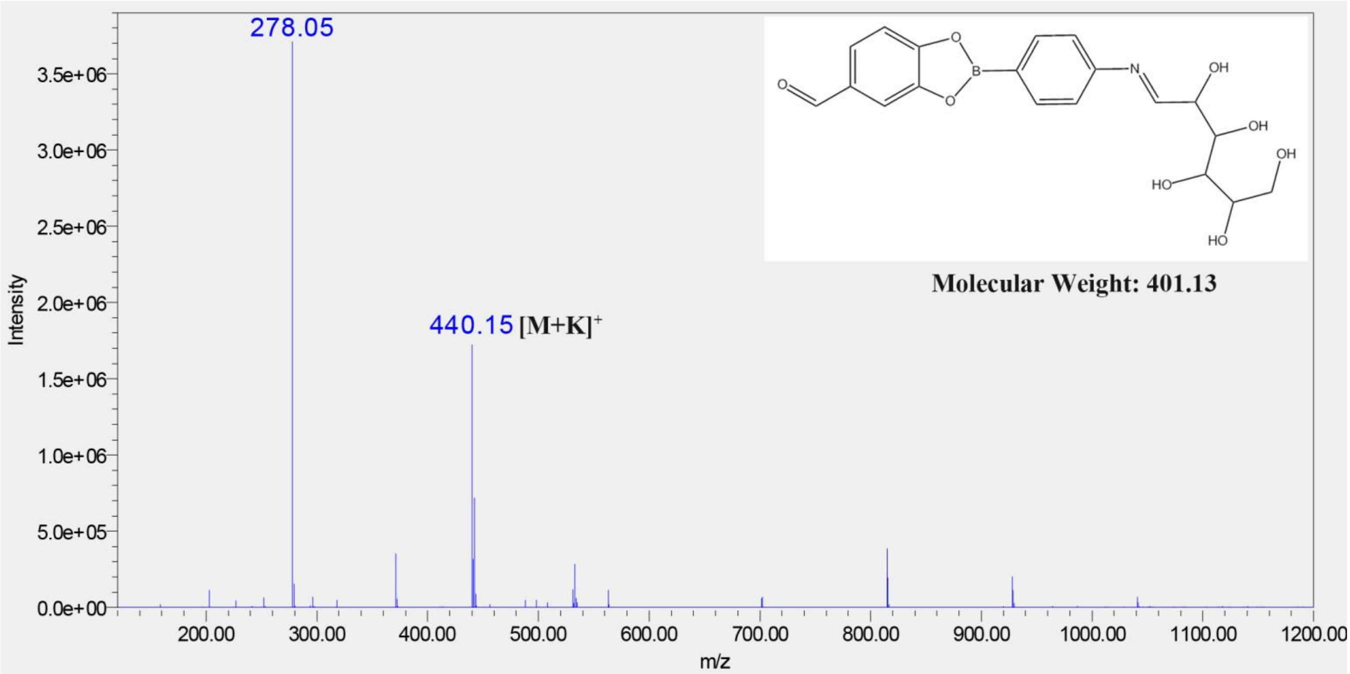


**Figure S3.** Mass spectrum and chemical structure of DPM. m/z = 440.15 ([M+K]$^+$) for DPM, m/z = 278.05 ([M+K]$^+$) for DP.

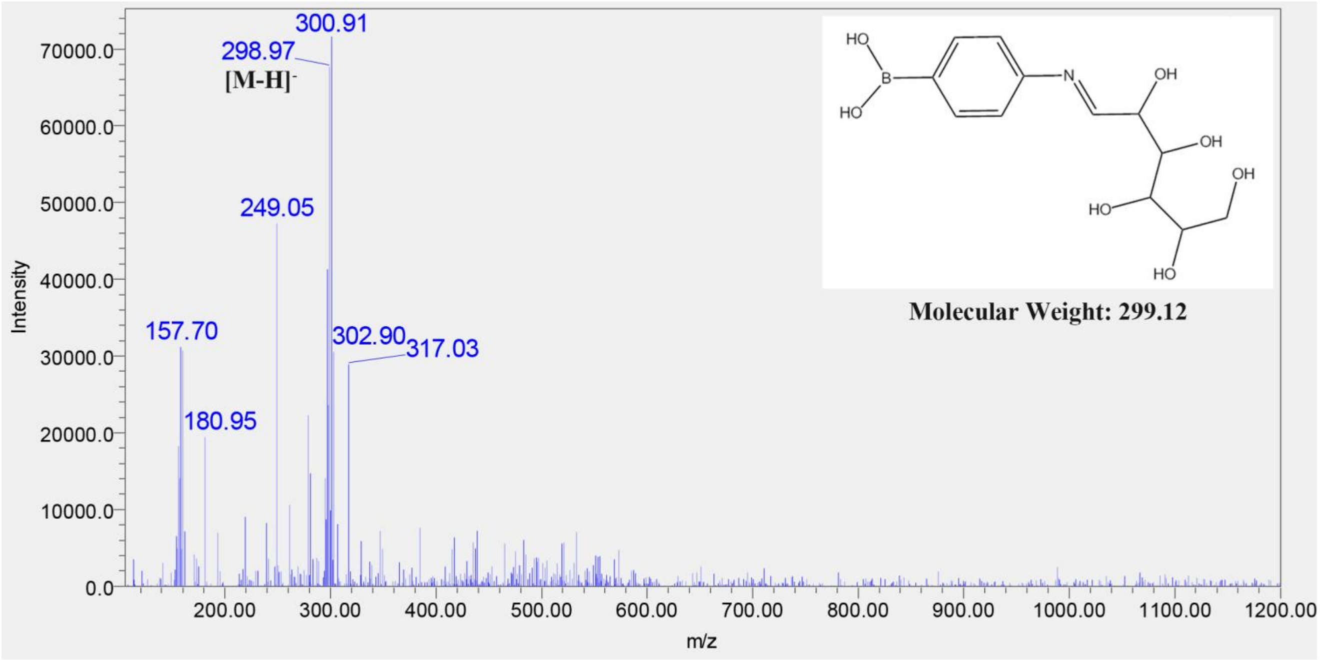


**Figure S4.** Mass spectrum and chemical structure of PM. m/z = 298.97 ([M-H]$^-$) for PM.

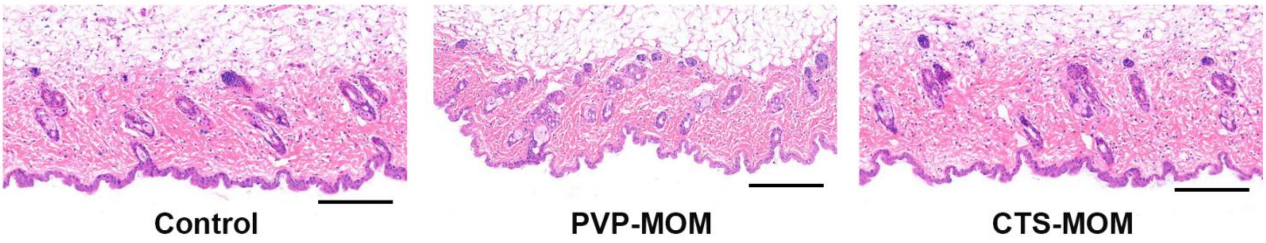


**Figure S5.** H&E-stained sections of mouse skin post-treatment with PVP-MOM and CTS-MOM. Scale bar, 200 μm.

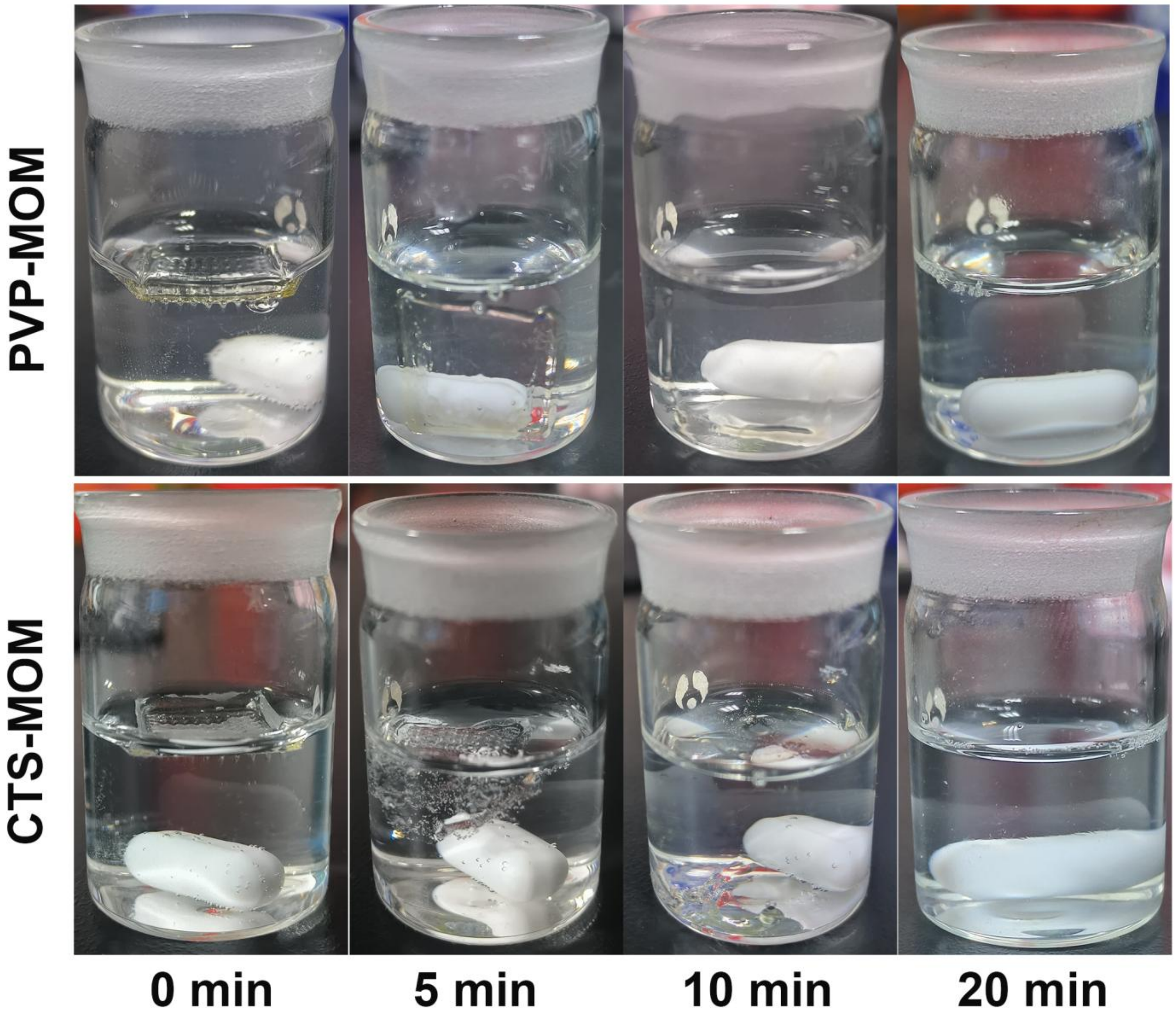


**Figure S6.** Images of MN dissolving in PBS at 37°C.

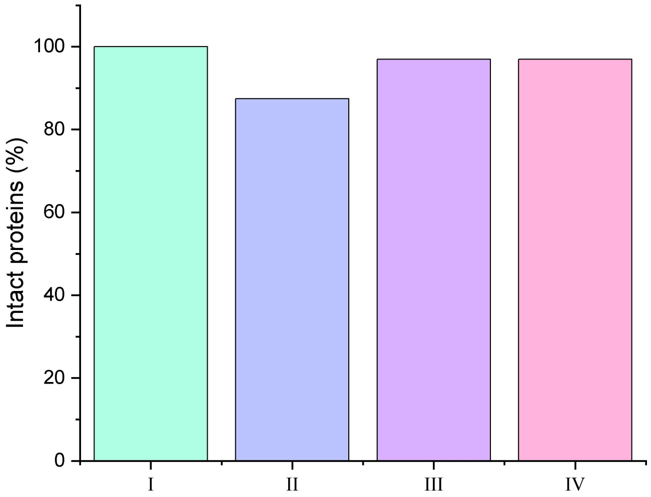


**Figure S7.** Percentage of the gray value relative to intact protein in Figure 4J. (I: OVA, II: OVA released from OVA/MSN-NH-DPM, III: OVA released from PVP-MOM, IV: OVA released from CTS-MOM).

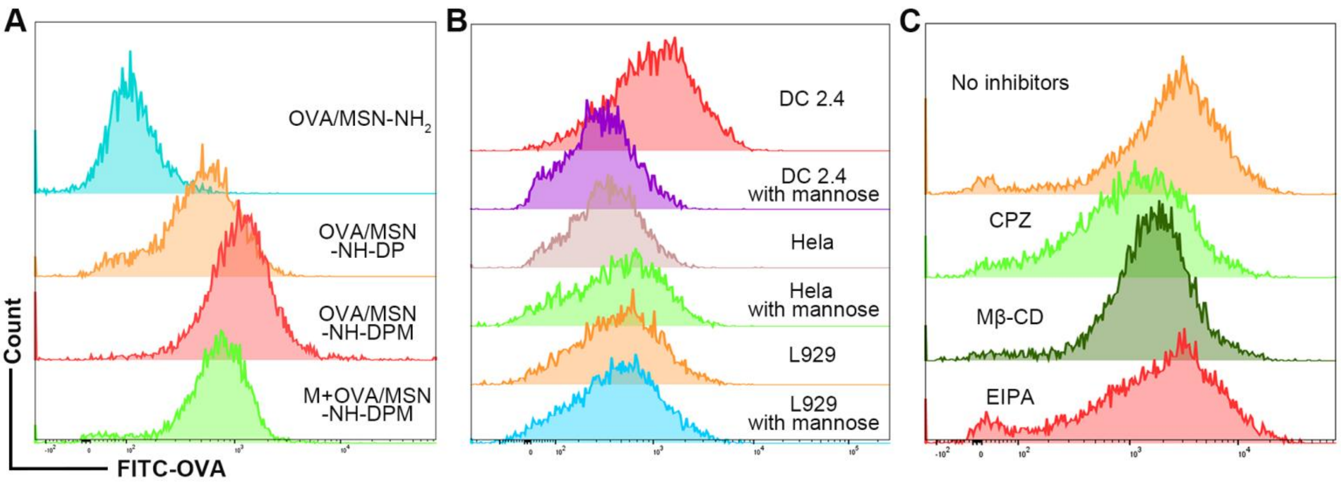


**Figure S8.** (**A**) Flow cytometer analysis for cellular uptake by DC 2.4 cells. (**B**) Flow cytometer analysis for competition of OVA/MSN-NH-DPM by adding mannose on three cell lines.(**C**) Flow cytometer analysis for endocytosis pathways of OVA/MSN-NH-DPM in DC 2.4 cells

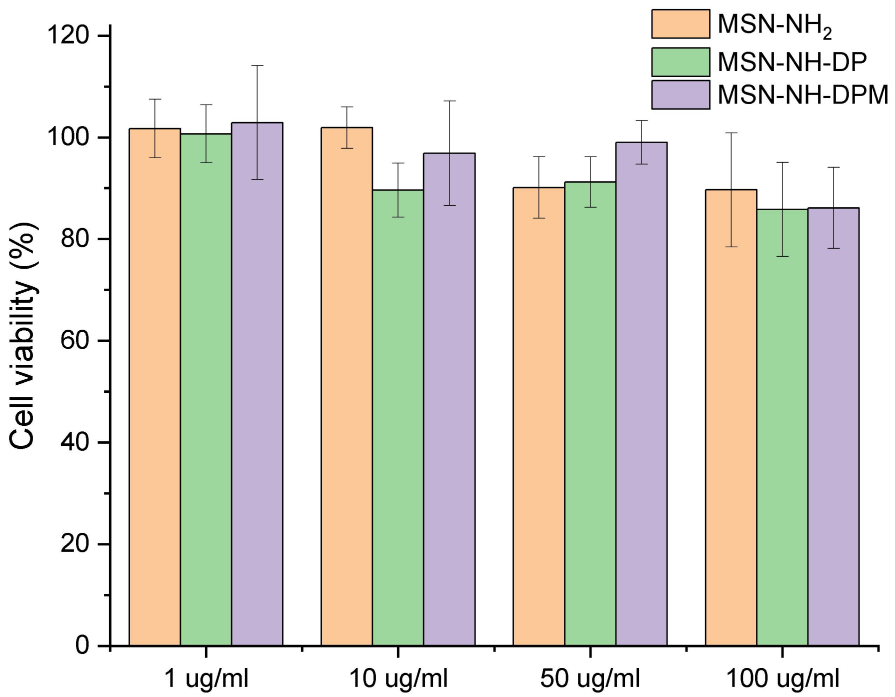


**Figure S9.** Cell viability of DC 2.4 cells exposed to different concentrations of MSN-NH$_2$, MSN-NH-DP and MSN-NH-DPM.

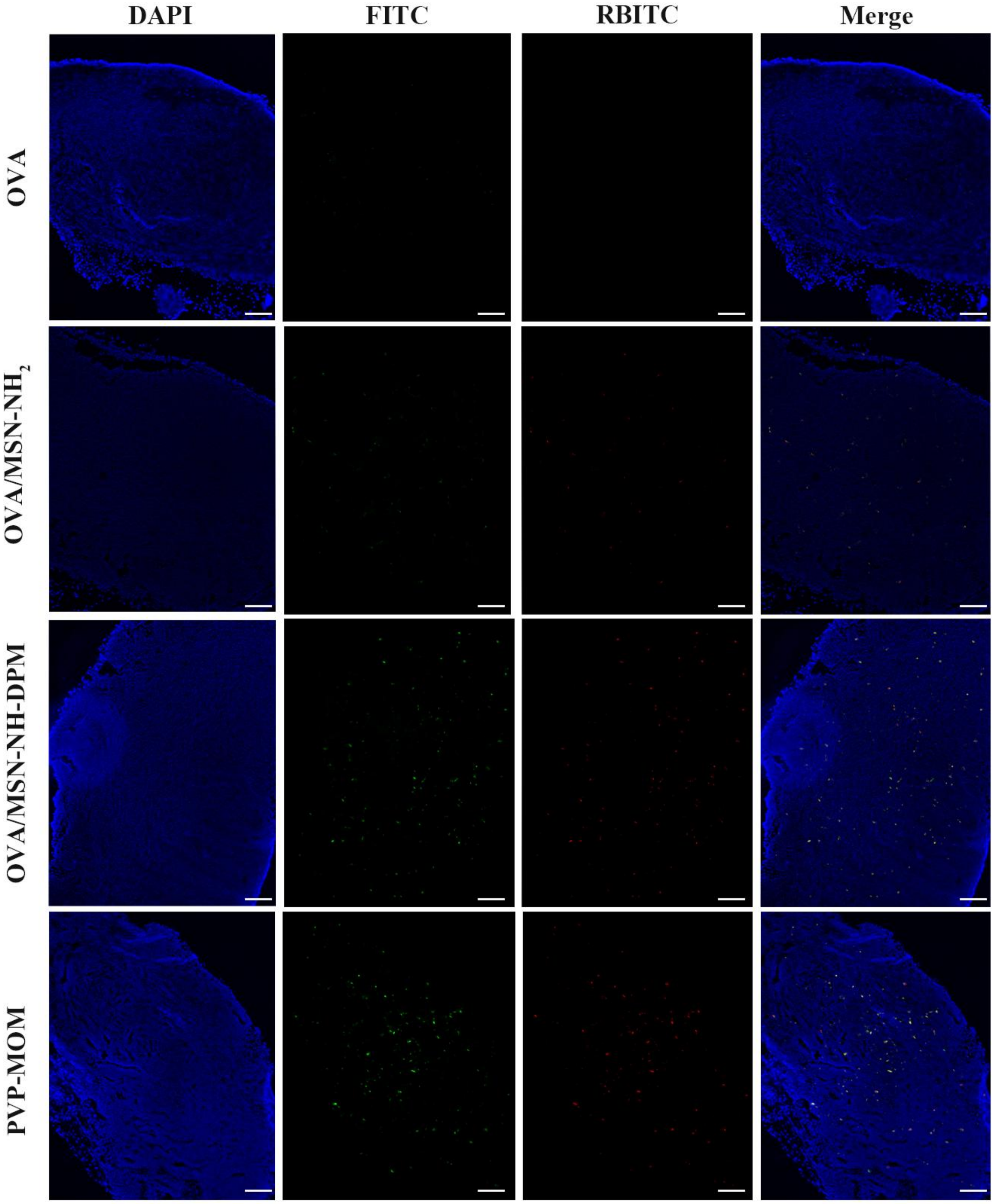


**Figure S10.** Colocalization images of FITC-OVA and RBITC-MSN after administered treatments with OVA (subcutaneously), OVA/MSN-$NH_2$ (subcutaneously), OVA/MSN-NH-DPM (subcutaneously), PVP-MOM in inguinal LNs tissue sections. Scale bars, 100 μm.

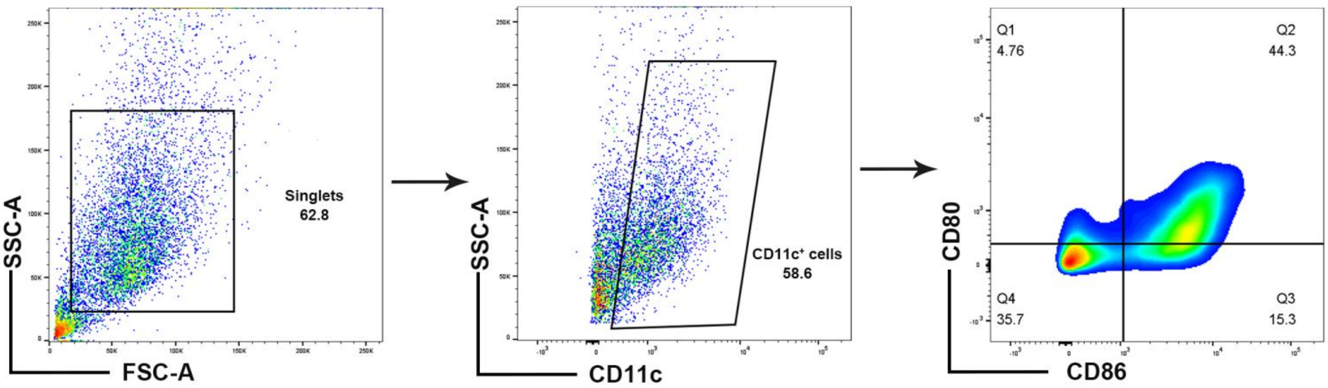


**Figure S11.** Gating strategy for flow cytometry assay.

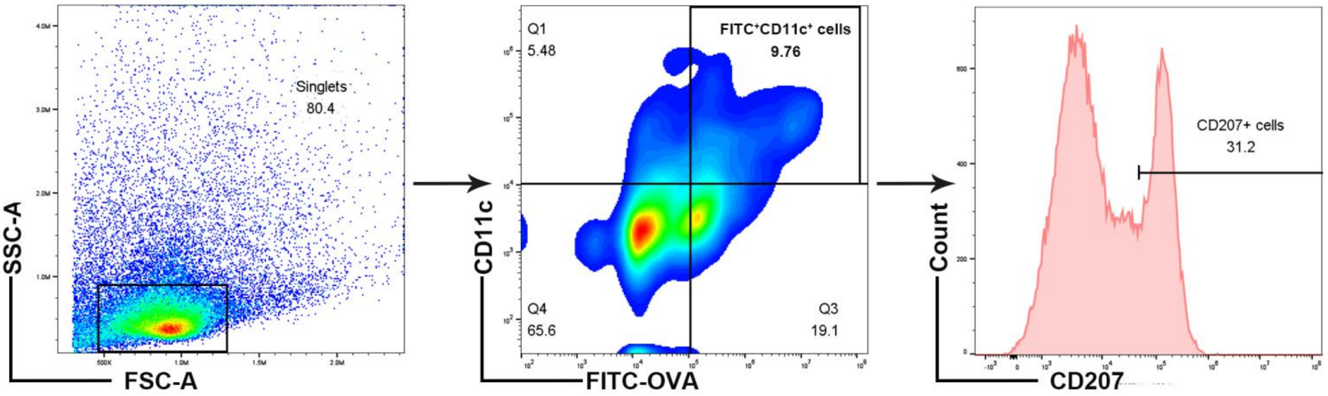


**Figure S12.** Gating strategy for flow cytometry assay.

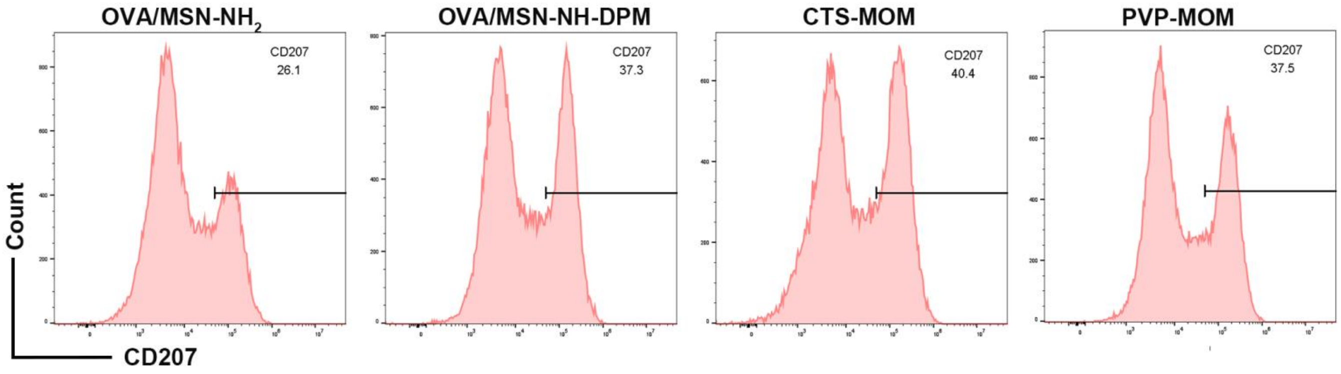


**Figure S13.** Representative flow cytometry plots of CD207$^+$ on FITC$^+$ CD11c$^+$ cells.

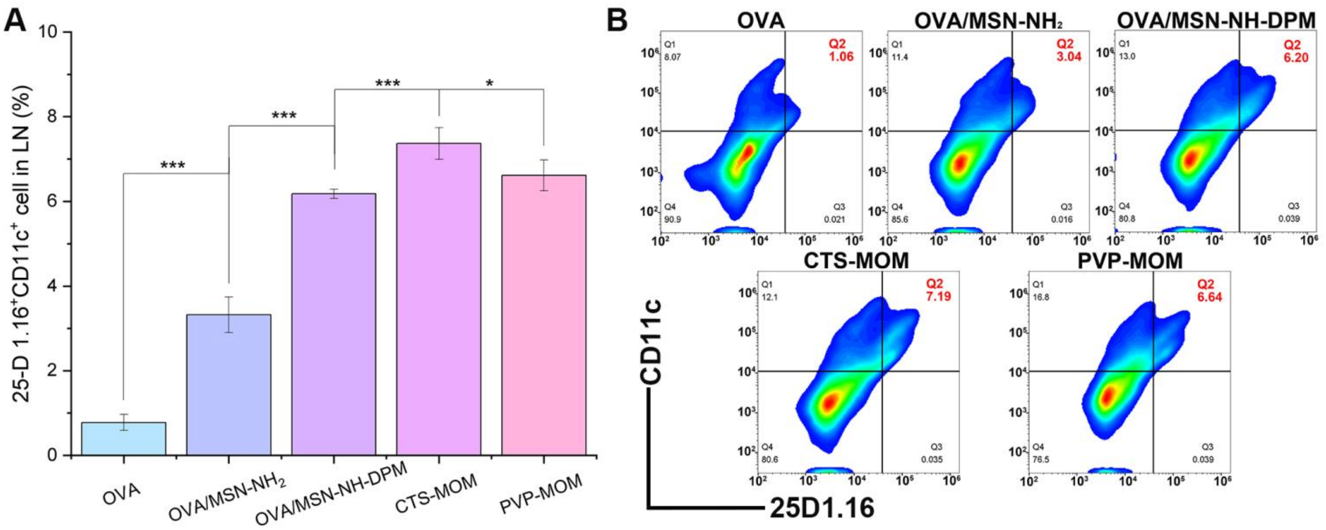


**Figure S14.** (**A**) The percentage of 25D1.16$^+$ on DCs (n=5). (**B**) Representative flow cytometry plots of 25D1.16$^+$ CD11c$^+$ cells.

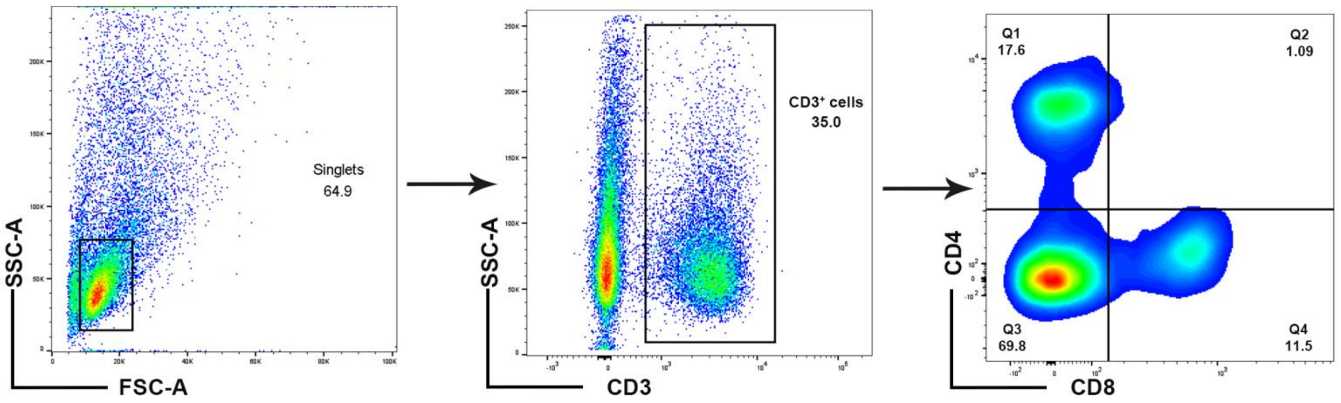


**Figure S15.** Gating strategy for flow cytometry assay.

## References


1. Fan N, Chen K, Zhu R, Zhang Z, Huang H, Qin S. et al. **Manganese-coordinated mRNA vaccines with enhanced mRNA expression and immunogenicity induce robust immune responses against SARS-CoV-2 variants**. Sci Adv. 2022;**8**:eabq3500.
